# Altered Metabolome of Lipids and Amino Acids Species: A Source of Early Signature Biomarkers of T2DM

**DOI:** 10.3390/jcm9072257

**Published:** 2020-07-16

**Authors:** Ahsan Hameed, Patrycja Mojsak, Angelika Buczynska, Hafiz Ansar Rasul Suleria, Adam Kretowski, Michal Ciborowski

**Affiliations:** 1 Clinical Research Center, Medical University of Bialystok, Jana Kilińskiego Street 1, 15-089 Bialystok, Poland; ahsanhameed@outlook.com (A.H.); patrycja.mojsak@umb.edu.pl (P.M.); adamkretowski@wp.pl (A.K.); 2 Department of Endocrinology, Diabetology and Internal Medicine, Medical University of Bialystok, 15-089 Bialystok, Poland; angelika.buczynska@umb.edu.pl; 3 School of Agriculture and Food System, The University of Melbourne, Parkville, VIC 3010, Australia; hafiz.suleria@unimelb.edu.au

**Keywords:** metabolomics, validated biomarkers, early biomarkers, diabetes mellitus, pre-diabetes, glucose intolerance, insulin resistance, obesity, gut microbiota

## Abstract

Diabetes mellitus, a disease of modern civilization, is considered the major mainstay of mortalities around the globe. A great number of biochemical changes have been proposed to occur at metabolic levels between perturbed glucose, amino acid, and lipid metabolism to finally diagnoe diabetes mellitus. This window period, which varies from person to person, provides us with a unique opportunity for early detection, delaying, deferral and even prevention of diabetes. The early detection of hyperglycemia and dyslipidemia is based upon the detection and identification of biomarkers originating from perturbed glucose, amino acid, and lipid metabolism. The emerging “OMICS” technologies, such as metabolomics coupled with statistical and bioinformatics tools, proved to be quite useful to study changes in physiological and biochemical processes at the metabolic level prior to an eventual diagnosis of DM. Approximately 300–400 such metabolites have been reported in the literature and are considered as predicting or risk factor-reporting metabolic biomarkers for this metabolic disorder. Most of these metabolites belong to major classes of lipids, amino acids and glucose. Therefore, this review represents a snapshot of these perturbed plasma/serum/urinary metabolic biomarkers showing a significant correlation with the future onset of diabetes and providing a foundation for novel early diagnosis and monitoring the progress of metabolic syndrome at early symptomatic stages. As most metabolites also find their origin from gut microflora, metabolism and composition of gut microflora also vary between healthy and diabetic persons, so we also summarize the early changes in the gut microbiome which can be used for the early diagnosis of diabetes.

## 1. Introduction

Diabetes mellitus (DM) is the most prevalent modern civilization disease and 8th major mainstay of mortalities globally. This worldwide pandemic of DM is associated with large financial strain on the healthcare systems of many countries, especially developing ones. The current projections from the International Diabetic Federations (IDF) predict that the number of diabetic patients will hit 629 million by 2045 and 34 million more people are at risk of developing DM than in 2015. Approximately 19 million more adult people are undiagnosed than in 2015 and 1 in 6 births are influenced by hyperglycemia in pregnancies [1]. Governments have allocated and spent almost USD 792 billion worldwide for the treatments of DM, out which almost 377 and 166 billion dollars were allocated and spent only by the US and European Union (EU) countries. In the developing countries the financial burden of DM treatment is mostly beard by patients themselves and it accounts for 25–40% of their monthly income. However, to make matters worse, 4 out of 5 people with diabetes live in low- and middle-income countries [1,2]. Therefore, new approaches are needed to lessen this financial pressure on both patients and healthcare systems which is achievable only by reducing the epidemic of DM.

To circumvent the onset and/or delay in the progress of DM, it is most effective to recognize the early stages of DM before major systematic damage (i.e., retinopathy, microvascular complications, nephropathy etc.) occurs. Currently, a number of laboratory-based-diagnostic-tools are available for the early detection of metabolic syndrome and related diseases which include impaired glucose tolerance (IGT), impaired fasting glucose (IFG), combined glucose tolerance (CGT) tests, anthropometric measurements and insulin sensitivity indexes [3]. These laboratory-based-tests are related to the (pre)diabetic state which may take years to proceed to chronic DM. The aforementioned diagnostic tests are directly related to glucose and insulin homeostasis. However, non-glucose/insulin related reliable and validated biomarkers are needed to complement our knowledge on diabetes development and indicate prognostic biomarkers. Regarding this, it has also been reported in the literature that metabolic syndrome (pre-diabetic/diabetic state) is likely to alter the metabolic pathways related to sugars, lipids, amino acids and their resulting metabolites resulting in the perturbed respective metabolites level in predisposed individuals compared to healthy ones [4]. These altered metabolite levels may serve as non-glucose/non-insulin related reliable and validated diagnostic biomarkers to recognize the pre-diabetic stage. Furthermore, many beneficial metabolites are produced as a result of intestinal microflora’s metabolism on sugars, lipids and amino acids. Therefore, metagenomic studies of gut microbiota have also been considered complementary to metabolomics studies to summarize the reported changes in the gut microbiota ecosystem.

A high throughput analytical technique such as metabolomics is immensely popular in epidemiological studies to provide the mining of these new reported biomarkers of disease risks and severity. This approach has the capability to detect the perturbation of one’s body metabolic pathways affected by disease and hence permit new insights into the physiological and pathophysiological development of disease [5]. The metabolomics technique has been used for screening potential diabetic patients for early diagnosis, prevention or delayed onset of type 2 diabetes mellitus (T2DM). Literature is full of many prospective, randomized, blinded, nested and case-controlled cohort studies where the populations of different geographical localities have been recruited and tested for decades to comprehensively understand the prognosis of DM in both vulnerable and non-vulnerable subjects. These studies came up with a wide range of early T2DM biomarkers, especially of sugars, lipids and amino acids origin. Most of these (pre)diabetes reporting biomarkers originating from sugars, lipids and amino acids metabolism are considered validated biomarkers. However, a few conflicting studies are also presented in literature and discussed in this review too. Most of these conflicts in findings may arise due to not considering varying covariates in research design and personal or communal genetic variations.

The data regarding the early biomarkers of DM (T2DM) in human beings is really dispersed and exhaustive. Therefore, the objectives of this comprehensive review were, for the first time in literature, to collect, compile and update as much reliable and validated early DM biomarkers data in one manuscript from published literature, which will be convenient for both physicians and potential DM sufferers to score their risk factors.

## 2. Metabolomics and Early Biomarker of Type 2 Diabetes Mellitus (T2DM)

The global prevalence of the T2DM pandemic has attracted wide attention due to its financial burden on health care systems. The failure in diagnosing prediabetes by conservative laboratory-based diagnosis tools at their latest stages could also be partially blamed for this pandemic. The laboratory-based diabetes diagnostic tools which are currently available include fasting blood glucose levels, insulin sensitivity indexes, oral glucose tolerance tests (OGTT) and glycated hemoglobin (HbA1c). It is estimated that up to 60% of cases of T2DM have never been diagnosed and/or misdiagnosed due to the sensitivity limitations of these assays at prediabetic and diabetic threshold levels [6]. Additionally, these trivial assays are not involved in staging the progress of diabetes as T2DM is considered a non-static condition and it keeps evolving from acute prediabetic (hypoglycemia, hyperosmolar hyperglycemic syndrome, ketoacidosis, and lactic acidosis) to chronic diabetic (diabetic coronary artery disease (CAD), cerebral vascular disease, diabetic retinopathy (DR), diabetic peripheral neuropathy, lower extremity vascular disease, diabetic nephropathy (DN) and diabetic foot disease) states [7]. The misdiagnosis and mistreatment of prediabetes and prediabetic complications has not only exposed people to non-essential medications with possible side effects but also to a source of economic loss. Preventing the onset of T2DM and/or diagnosing the early stages of diabetes followed by respective targeted treatment is the most economical way to treat T2DM before the occurrence of systematic damage and chronic complications. Apparently, the failure of the diabetic diagnostic tools currently available to diagnose prediabetes makes the search inevitable for new biomarkers/predictors to complement the current diagnostic measures. Recently, the inclusion of low plasma adiponectin concentration as a strong predictor for future T2DM development [8] further suggests the significant scope of setting the complementary biomarkers of T2DM risk. Moreover, highly sensitive and specific biomarkers are urgently needed in order to early diagnose T2DM. Metabolomics provides a great opportunity to indicate these novel biomarkers.

Metabolomics has been increasingly used in epidemiological studies for unveiling the novel association between metabolic pathways and disease. It is referred to a systematic study of identification and quantification of low molecular-weight metabolites in a given biological sample. The pool of these metabolites plays an important physiological role in the biological systems and is considered a promising candidate for studying disease phenotype as disrupted levels of metabolites were found in prediabetic individuals [9]. Two major platforms are used in metabolomics research, i.e., mass spectrometry (MS) and nuclear magnetic resonance (NMR) spectroscopy. MS, the most frequently employed approach, is high-throughput and sensitive but destructive in nature. It is often used in combination with separation techniques such as (high/ultra-pressure) liquid chromatography, gas chromatography and capillary electrophoresis. NMR, on the other hand, is non-destructive, robust and reproducible with minimal sample preparation, separation and ionization steps. As no single platform exists to capture the global profile of the whole metabolome, a multiplatform approach is mostly applied to get an all-inclusive understanding of metabolic variations and widen the “window” of significant metabolic discrepancies [10]. The application of metabolomics in T2DM studies has been in progress for the last two decades with the successful provision of novel insights into the pathophysiological mechanism and metabolic profiling. These studies have come up with the identification of novel biomarkers related to insulin resistance and T2DM biomarkers [3,4,10]. It is highly probable that a growing number of these novel T2DM biomarkers can be translated into clinical applications that will upgrade the current medical routine in regards to personalized medicine.

## 3. Biomarkers of Disturbed Protein Metabolism

Protein and glucose metabolism are tightly linked and accordingly regulated at metabolic and molecular levels. Dietary and endogenous amino acids (AA) relate to glucose metabolism via gluconeogenesis. The catabolic breakdown of AA provides the fuel for gluconeogenesis. The deamination of AA forms ketoacids such as oxaloacetate and pyruvate which feed the gluconeogenesis [11,12]. On the other hand, AA are also de novo biosynthesized from the Krebs cycle-derived carboxylic acid-intermediates by transamination. These free AA modulate the AA-type-dependent glucagon and insulin secretion and hence glucose metabolism [13]. In short, AA are not only an energy reservoir for gluconeogenesis, but their de novo biosynthesiz influences the glucagon and insulin release. In prediabetes, the glucagon not only over-activates the gluconeogenesis in the liver and kidneys but also disrupts the de novo biosynthesis of AA, which makes the AA good candidate biomarkers. In the meantime, many studies have claimed a positive association of branched-chained AA (BCAA), aromatic AA and other AA with the risk of developing T2DM as follows.

AA: Elevated (serum/plasma) AA level is an important early biomarker of glucose intolerance, insulin insensitivity and, subsequently, diabetes. Many epidemiological cohort studies have witnessed an increased level of AA in newly diagnosed diabetic patients in the follow up investigations hinting towards an impaired glucose/hexoses metabolism [14]. An increased serum AA level reduced the insulin sensitivity and uptake of hexoses by offering competition at the substrate level to glucose oxidation and also by interfering with insulin signaling [11,13]. Higher serum AA levels were found to diminish the peripheral uptake of hexoses along with the suppression of endogenous glucose production (EGP) and body glucose disposal by 25% [15]. Similarly, the rate of glycogen synthesis was recorded to reduce up to 64% with a rise in serum AA levels under insulin stimulated conditions which accounted for AA modulated reduced glucose absorption in the body. This decline in glucose absorption is accompanied by the down-regulation of glycogen synthase, glucose transporter, phosphorylations and G6P [11,16]. Many studies have recorded this increased AA level as a future risk factor of developing T2DM. Most of these AA not only belong to BCAA but also to aromatic and aliphatic AA. In a closely matched case-control study, three aromatic (phenylalanine, tyrosine, and tryptophan) and one aliphatic AA (lysine) were found to be associated with the future risk of T2DM. The predictive power of lysine was also viable after the OGTT while comparing cases vs. controls. The additional stratified analysis in follow-up duration recorded the retainability of the predictive power of these AA up to 12 years from the baseline. For each increase in the SD of these AA, the odds of future T2DM development increased by 57–102%, further signaling towards a 2- to 3.5-fold higher probability of developing T2DM in top quartile individuals. The conditional regression analysis with isoleucine, phenylalanine and tyrosine also reported a five- to seven-fold higher probability of developing T2DM in top quartile individuals [17]. In the Insulin Resistance Atherosclerosis (IRAS) cohort, the individuals of four ethnicities (European-American, Hispanic, and African-American) who converted to T2DM in their follow-up of five years recorded similar results. A distinctive metabolome profile was noted in T2DM-converted subjects compared to healthy controls with significantly higher concentrations of phenylalanine, tyrosine, combined glutamine and glutamate, and valine associated with insulin resistance (Table 1). An 11–15% increase in these AA was seen whereas a 22% lower level of glycine was noted in highly insulin resistant individuals. A nominal difference was also noted between high/low insulin-resistant persons to T2DM-convertees. Alanine and aspartate/asparagine levels increased in the T2DM-converters only compared to high/low insulin resistant persons [18]. The SABRE (Southall and Brent Revisited) cohort study comprised of non-diabetic South Asian migrants in Europe/UK pinpointed nine AA in relation with the biomarker of obesity and insulin resistance in a follow up of 19 years. This study also described a significant positive correlation of phenylalanine, tyrosine and alanine, a weak positive relationship of histidine, and a significant negative correlation of glutamine and glycine with insulin resistance and glycemia [19]. In another Asian-Japanese cohort study, the authors measured the level of plasma-free AA (BCAA and aromatic AA) in correlation with obesity and diabetes and was able to predict the future diabetes risk in a minimal time of four years. This study further cited the negative correlation of clusters of glycine, serine, glutamine, and asparagine with obesity, body mass index (BMI), insulin resistance and 120 min insulin resistance assay. The increment of 1 SD of the odds ratios of the plasma-free AA increased the future risk of T2DM, metabolic syndrome, dyslipidemia or hypertension by 2.06%, 3.04%, 1.98%, and 1.42% respectively [20]. The longitudinal, nested and cross-sectional studies from two Chinese cohorts, Shanghai Obesity Study (SHOS) and Shanghai Diabetes Study (SHDS), also noted increased serum levels of aromatic AA at baseline in those individuals who develop T2DM in a follow up of 10 years [21]. The Finnish cohort study of 9369 nondiabetic or newly diagnosed T2DM Finnish men, namely population-based Metabolic Syndrome in Men (METSIM) also cited increased concentrations of tyrosine, glutamine and alanine in a 4.7-year follow-up [22]. An aliphatic AA called 2-aminoadipic acid was also found to be an early biomarker for T2DM risk. The degradation of lysine usually results in the 2-aminoadipic acid that may serve as a substrate for tryptophan catabolism. A strong association of 2-aminoadipic acid with T2DM risk was for the first time cited by Wang et al. [23]. The fasting plasma levels of prediabetic patients were found to be high in this amino acid. Following adjustments for age, sex, BMI and fasting conditions, the conditional logistics regression models noted 60% odds of future T2DM risk after each standard deviation (SD) increment of a logged biomarker. The twelve years-follow up-period showed 4-fold higher odds of developing T2DM in the top quartiles of plasma 2-aminoadipic acid concentration. The adjustment of data with respect to parental history, dietary habits, lifestyle, fat/protein/carbohydrate intake and total caloric intake did not bring about any variations in this risk factor [24,25]. The independent work on Malmo diets and cancer studies also served as a replication of these results, which also indicated a 57% rise in the T2DM risk for each increment of SD of 2-aminoadipic acid concentration. Aromatic and branched-chain amino acids are also biomarkers of incidence of T2DM, but recent studies have not found any correlation between 2-aminoadipic acid and aromatic or branched-chain amino acids. However, a modest association of 2-aminoadipic acid with lysine, kynurenic acid and anthranilic acid was observed [16,23]. The Dongfeng–Tongji (DFTJ) and Jiangsu non-communicable disease (JSNCD) independent nested case control cohort models were also used to predict the identified metabolites using the traditional risk factors [26]. Qiu et al. [26] identified 52 metabolites, among which 20 AA were found to have associated positively with DM risk in both models. The dietary variables also did not change the four AA biomarkers out of 20 (i.e., alanine, phenylalanine, tyrosine and palmitoylcarnitine) which had a false discovery rate correction (FDR) < 0.01. The exploratory analysis of the pooling of other metabolites also identified an additional 12 metabolites, including such AA as glutamate, betaine, ornithine, leucine/isoleucine, valine and proline which achieved FDR < 0.01. The four metabolites i.e., phenylalanine, alanine, palmitoylcarnitine and tyrosine were found consistently associated with the risk of T2DM. Another aliphatic AA called alanine is a hepatic substrate and stimulator for gluconeogenesis and glucagon secretion and its circulating amount was also found to be a predictive metabolite of T2DM risk in many cross-sectional and prospective studies [4,27]. Elevated levels of alanine have already been found to have a positive association with T2DM in Finland, UK, and South Asia populations, as stated above. Moreover, the evident relationship of phenylalanine and tyrosine with the risk of T2DM was also significant due to an increase in insulin resistance through blocking the transport/phosphorylation of glucose. In the case of tyrosine, it is a far more powerful indicator of T2DM risk in South Asians [27]. The population-based KORA (Cooperative Health Research in the Region of Augsburg) and European Prospective Investigation into Cancer and Nutrition (EPIC)-Potsdam cohort study identified five baseline metabolites out of 131 (using pairwise comparison, multivariate (logistic) regression analyses followed by non-parametric random forest and the stepwise parametric regression) specifically associated with the pre-diabetic conditions to examine their ability to forecast the pre-diabetic conditions earlier [4]. The non-parametric random forest and the stepwise parametric regression recorded glycine, (lysophosphatidylcholines (LysoPC) (18:2), LysoPC (17:0), LysoPC (18:1) and C2) as novel candidate biomarkers of T2DM. In pursuit of establishing the predictive values of these metabolites, the baseline concentration of these metabolites in the KORA cohort (118 incident and 471 healthy controls) were compared, which revealed significant differences only for glycine and LysoPC (18:2). Each increment in the standard deviation of these two metabolites associated with a 33% less risk of future diabetes. The individuals in the fourth quartile were at three times less risk of diabetes than people whose serum glycine and LysoPC (18:2) levels were at first quartile. Therefore, the baseline decreases in the serum levels of glycine and LysoPC (18:2) were cited as powerful indicators of the future onset of diabetes [4]. Later on, the replicative prospective study with EPIC-Potsdam cohort also reported similar results using targeted metabolomics citing an increased phenylalanine concentration and reduced glycine concentration as biomarkers for future T2DM occurrence [28]. Earlier, the work of Pontiroli et al. [29] also confirmed a low level of glycine as a result of progressive insulin resistance which, in turn represses, the expression of ALAS-H catalyzing the conversion of glycine and succinyl-CoA into 5-aminolevulinic acid. In short, most of the early diabetes biomarker mining studies find AA as early predictors of T2DM. In summary, with the exceptions of glycine, serine, asparagine and histidine, most of studies stated increased concentrations of AA as a risk predictor of the future onset of T2DM. The predicting power of different AA may vary depending on the early diabetic stage, ethnicities and genetic background.

BCAA: the first report of BCAA (i.e., valine, isoleucine, and leucine) correlation with insulin resistance, impaired insulin signaling and diabetes appeared in 1970 [30]. Since then, an overwhelming number of published data have advocated the predictive and pathogenic relationship of increasing plasma BCAA concentration with obesity, insulin insensitivity and diabetes. The literature assertions of higher plasma BCAA levels are mainly due to the concept of insulin resistance resulting from the activation of the rapamycin molecular target (mTOR). It is currently poorly understood which mechanisms are involved in increasing the BCAA level. However, most of the mechanistic work on this topic cited the downregulation of mitochondrial branched-chain keto acid dehydrogenase (BCKDH) and branched-chain aminotransferase (BCATm) expression followed by under-transamination and thereafter under-decarboxylation and under-dehydration of BCAA [31]. Many in vitro and in vivo studies stated that increasing BCAA (especially leucine) encouraged the insulin resistance by the activation of mTORC1 and S6 kinase followed by the phosphorylation of insulin receptor substrates S1 and S2. The deprivation of individual BCAA can promote the activity of mTORC1/S6K and adenosine monophosphate-activated protein kinase (AMPK) signaling pathways resulting in improved insulin sensitivity [32,33]. The prospective roles of reporting biomarker BCAA + aromatic AA in the pathogenesis of T2DM were further investigated in a Finnish cohort study stating the BCCA + aromatic AA as an early predictor of insulin resistance in young Finnish adults after 6 years of follow-up study [34]. A recent cohort study on 3000 volunteers found BCAA to be a valid indicator of the future risk of DM [20]. Various prospective, case-controlled and nested studies on the subjects of different ethnicities found elevated levels of BCAA in the pre-diabetic, insulin-resistant and T2DM subjects [35,36]. The longitudinal nested control-case study conducted on UK Caucasians found three BCAA as early predictors of T2DM risk [17]. A 12-year follow-up study found 2.5- to 3.5-fold higher odds of T2DM risk in the top quartiles of individuals as compared to those individuals whose plasma amino acids were in the lowest quartiles. The adjustments of predictive models for parental history, dietary variations and serum triglycerides further elevated the odds ratios of metabolites, especially amino acids. The replication analysis with Malmo and cancer diets also established a substantial relationship of four amino acids (leucine, tyrosine, valine, and phenylalanine) with incidence of diabetes. In Malmo diets, the three amino acid combination (isoleucine, tyrosine, phenylalanine) quadrupled the incident of diabetes compared to lowest quartiles [17]. Tillin et al. [19] conducted a multi-ethnic cohort study for a 19 year period of follow-up and witnessed that 14–35% of the population who had shown higher BCAA level at baseline developed T2DM. The logistic regression results of this study clearly bespoke about the obvious involvement of BCAA (odds ratio (OR) = 3.14 to 3.36) in the development of diabetes. Chen and his colleagues also reported that after 10 years of follow-up, a higher positive correlation was detected between the baseline five BCAA and incidence of DM in the understudied Chinese population [37]. The seven years of follow up in the EPIC-Potsdam cohort study found a positive correlation between future DM risk and valine, isoleucine and leucine [28]. A similar study was also performed on the Chinese Han ethnic folks who are among the highest diabetes vulnerable group [38]. This study verified the prediction-ability of a chosen model and proposed higher levels of alanine, lactate, β-hydroxybutyric acid, phosphate, leucine, α-hydroxyisobutyric acid, serine, isoleucine, palmitic acid, pyroglutamic acid, stearic acid, oleic acid, 1-monopalmitin, arachidonic acid and 1-monostearin while substantial lower levels of 2-ketoisocaproic acid as early biomarkers of T2DM. Similarly, Fiehn et al. [35] noted a 50% higher concentration of plasma leucine with a 27% higher amount of its catabolic secondary metabolite called 2-ketoisocaproic acid (α-ketoisocaproate). The mean concentration of plasma valine was 20% higher in pre-diabetic weight/age matched African-American ethnical subjects. The enrichment of the plasma AA pool with valine and leucine is also strongly correlated with the plasma acetyl-carnitine concentration. The AA score (sum of BCAA + aromatic AA) was found to be in association with baseline insulin resistance/HOMA-IR even after the adjustment for metabolic factors. The magnitude of this association was found to be more pronounced for men than for women. The authors stated that both the BCAA + aromatic AA were strong predictors of insulin insensitivity in men, whereas only valine, leucine and phenylalanine showed a positive correlation for HOMA-IR in women [22,35,39]. Stancakova et al. [22] observed that among all BCAA, isoleucine was found to be strongest and most reliable predictor of insulin resistance. Some studies concluded that fasting plasma BCAA levels are a reliable predictor of T2DM, whereas circulating BCAA correlated positively with the indices of uncontrolled blood glucose and insulin resistance in overweight individuals [40]. Some studies also documented the correlation of plasma BCAA is only significant in prediabetic obese subjects who started losing significance upon losing weight and improving insulin sensitivity [41]. Other work has also stated the BCAA is solely responsible for insulin resistance, at least under the circumstances of high tissue FA availability and in high-fat feeding conditions [33]. With respect to age, no obvious difference was noted in the correlation of BCAA with insulin resistance between youth and adults’ subjects [42]. Although an ethnicity based studies came up with some mixed conclusions. The cohort studies on the Asian population experienced and supported BCAA as a validated biomarker for the prognosis and development of T2DM [19,43,44], whilst, at the same time a predictive model study in American-Indians failed to developed a reporter BCAA notion [45]. The difference in the predictability of BCCA for the future risk of DM in different ethnicities (Caucasian Hispanic and Africans Americans) was further investigated by Lee et al. [46] and dictated that diabetic risk was more prevalent among the Hispanic Caucasians compared to African Americans. Chen et al. [21] recently conducted plasma metabolomics on the subjects recruited from the SHDS cohort study in which they identified increased (2-folds) BCAA even at baseline between the future diabetic patients and the healthy controls. The fitting of basic and advanced regression models to these BCAA metabolites with 3–14 confounding factors verified the correlation of BCAA with the future risk of diabetes without any dependence on physical activity factors. The discrimination between the healthy and diabetic groups was also evident in the area-under-curve (AUC) interpretations of these BCAA. As expected, the regrouping of diabetic and healthy controls also exhibited a strong relationship of BCAA with diabetes in diabetic people with fold change (FC) >2 and odds ratio (OR) >1.5 [21]. Subsequently, the correlation of the worsening of metabolic control of glucose with the BCAA was investigated, which was important to confirm further the BCAA status of a validated early biomarker of diabetes. The authors used the UCD-T2DM rat models, which were homozygous for β-cell defects with diabetes-prone obese ancestors [41,47]. Piccolo and his colleagues asserted that metabolites of BCAA are more robust markers of insulin resistance than BCAA themselves. 2-ketoisocaproate and 2-hydroxybutanoic, the metabolites of leucine and methionine/cysteine respectively, have been implicated as the most reliable markers of diabetes risk as these metabolites were lower before the onset of insulin resistance and their level increased just after 3 weeks of detected metabolic insulin resistance. Besides BCAA, all other gluconeogenic and ketogenic amino acids (i.e., alanine, glycine, methionine, serine, thrionine, trypsine and ornithine) were found to have been reduced by 16–36% in the pre-diabetic UCD-T2DM rat model. The plasma BCAA correlation with total fasting plasma glucose, adiposity and insulin become more significant with the worsening of the metabolic control of glucose [41,47]. In addition to the work of Piccolo et al. [41,47], many metabolomics studies also established the fact that catabolic products of BCAA also have equal predictive qualities to BCAA. The BCAA-derived short-branched fatty acid and branched-chain keto acids are new to this list. The odd carbon number acylcarnitines, another BCAA breakdown products, are also considered as the latest reported biomarker of insulin resistance development [48,49]. However, the only disadvantage with BCAA-derived metabolites is having a lower plasma/tissue concentration and stability than BCAA, resulting in higher analytical variations. The intake of BCAA with a high-fat diet for 9–16 weeks increased (2.3- to 3.1-fold) subcutaneous/visceral fat mass and respiratory quotient (RQ), HOMA-IR in obese Wister rats followed by up-regulation of 14 energy metabolism-related hormones (glucagon-like peptide-1 (GLP-1), amylin, pancreatic polypeptide, resistin and insulin-like growth factor binding protein-3-IGFBP-3). The evaluation of BCAA related metabolites and HOMA showed a linear correlation, which signified the BCAA responsible for obesity-related morbidities. Like HFD, BCAA diets contributed equally towards the development of insulin resistance as HFD, HFD + BCAAA and BCAA diets impaired the phosphorylation of AKT/protein kinase B in individual experiments. A BCAA rich diet equally raised insulin resistance and weight gain like HF diets, but the addition of BCAA in HF diets reduced food intake [32,50]. Please note that diabetic and obesity-promoting effects of BCAA are only possible when all three BCAA were used in combination and supplementation of single BCAA usually does not increase the insulin resistance [51]. Many studies documented a reduced level of other AA after the increase of BCAA and aromatic AA. In this context, the most favorable idea is that higher BCAA plasma levels (in insulin-resistant cases) markedly reduce the catabolism in main tissues which, in turn, limits the obligatory AA concentrations in tissues considered responsible for normal metabolism. There are also many reports in the literature supporting this school of thought and indicating the reduced expression and activities of two initial catabolic BCAA enzymes (branched-chain amino acid aminotransferase-BCATm branched-chain a-ketoacid dehydrogenase-BCKD) in the liver and adipose tissue. T2DM patients also showed a 20% less whole-body clearance of BCAA [52,53]. Literature has also shown the declining outcome of the impairment of BCAA oxidation or turnover in T2DM or obese people [54]. In fact, additional work is needed to find the exact reasons for elevated levels of BCAA (if any) and to track down the fate of proteins and BCAA metabolism in diabetic and obese subjects. The plasma biotin status is also crucial for catabolic carboxylation enzymes of cysteine, TCA cycle anaplerosis and BCAA. Low biotin levels were found in insulin resistant patients with higher 2-hydroxybutyric acid (2-HB) metabolite. The higher 2-HB concentration induced the dysfunctional bioactivity of biotin tissues which in turn affects the cysteine/BCAA/TCA cycle anaplerosis metabolism [35]. In summary, the plasma concentration of BCAA has a positive relation with the future risk of T2DM. The comparative predictive-power of BCAA may vary with leucine and isoleucine cited as relatively the most powerful reporter BCAA biomarker of T2DM. The level of BCAA enabling this group of metabolites to be considered as a reliable biomarkers needs to be defined with respect to sex and ethnicities. The intake of BCAA could pose serious health problems in individuals with unknown insulinemia, dyslipidemia and glycemia.

## 4. Biomarkers of Disturbed Lipid Metabolism

Homeostasis of lipid metabolism is a tightly regulated act at various molecular and cellular levels in healthy subjects whereas obesity is the central risk factor for disturbances in homeostasis of lipid metabolism (hence T2DM pathogenesis) leading to the accumulation of excess fat, dysregulated glucose and lipid metabolism, impaired insulin and adipocyte signaling, and various other pathologies related to cardiovascular disease, arthrosclerosis and inflammation. This alternation in the lipid metabolism at the cellular level usually occurs years before the diagnosis of diabetes. Many studies in the literature cited this varied window-period from altered lipid metabolism to a final diagnosis of T2DM in many cohort studies related to biomarkers. These biomarkers belong to dynamic classes of lipids but we, for the convenience of readers, will state only those signature early biomarkers of T2DM risk which belong to the three main classes of lipids.

**Figure 1:**
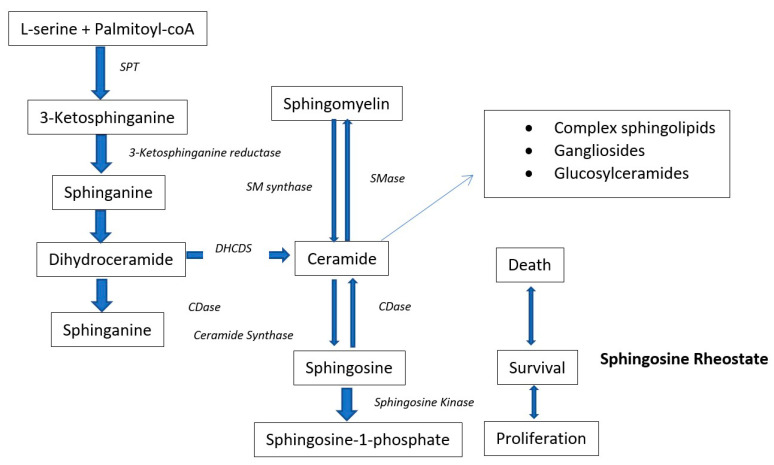
Schematic presentation of the de novo biosynthesis of ceramide and ceramide metabolism. SMase, sphingomyelinase; CDase, ceramidase; SM synthase, sphingomyelin synthase. (Modified from: Brozinick, Hawkins [87]).

Glycerolipids and phospholipids (PL): high-density-lipoprotein-cholesterol (HDL-C), low-density-lipoprotein-cholesterol (LDL-C), triglycerides (TG), total cholesterol (TC) and BMI are the typical dyslipidemic risk factors/biomarkers for T2DM. However, recently, several cohort studies focused on the lipidomics of the subjects for finding novel biomarkers of T2DM. All these lipidomics studies found varying quantities of diacyl phosphatidylcholines (PC), glycerophospholipids (GPL), phosphatidylethanolamines (PE), alkylacyl phosphatidylcholines (PC), lysophosphatidylcholines (LysoPC), alkylacyl phosphatidylethanolamines (PE), triacylglycerols (TG), lysophosphatidylethanolamines (LysoPE), sphingomyelins (SM), cholesterol esters (ChoE) and ceramides (Cer) in the (pre)diabetic compared to non-diabetic patients [3,4,55,56,57,58,59]. These lipid metabolites are diabetic risk predictors in human beings. To investigate the mechanism by which these lipids contribute to the prediction of diabetic risk, many studies compared the lipidome of (pre)-diabetic persons with healthy controls in several cohort studies with a follow up of ≥5 years. A prospective case-control cohort study on European Caucasians identified 34 metabolites significantly associated with the early risk of T2DM. The risk of T2DM was positively associated with phenylalanine, hexose, and diacyl-phosphatidylcholines (36:1, 32:1, 40:5 and 38:3), while an inverse relation was detected with sphingomyelin (16:1), glycine, acyl-alkyl-phosphatidylcholines, lysoPC (18:2) as well as PC (34:3, 42:5, 40:6, 44:5 and 44:4) (Table 1) [28]. The unsupervised PCA divided the metabolites into two metabolic factors. Metabolite factor 1 (i.e., sphingomyelins, acyl-alkyl-phosphatidylcholines and lysophosphatidylcholines) reduced the incidence of T2DM by 69% and metabolite factor 2 (i.e., BCAA, diacyl-phosphatidylcholines, propionyl carnitine, aromatic amino acids, and hexose) increased the risk of T2DM almost 4 times. [28]. Some metabolites of phospholipid metabolism (lysophosphatidylcholine C18:2, acyl-alkyl-phosphatidylcholines, and glycine) were found in hyper-insulin-sensitivity cases whereas some phospholipid metabolism metabolites (e.g., diacyl-phosphatidylcholines, acyl-alkyl-phosphatidylcholines, sphingomyelin C16:1) were associated with insulin resistance and less insulin secretion. It is worthy to note that choline derived phospholipids were significantly associated with the risk of T2DM [60,61]. These kinds of phospholipids also acted as a potent antioxidant to prevent the oxidation of lipoproteins and also required for the secretions of VLDL and VHDL from hepatic tissues [62]. These choline derived phospholipids are in a positive relationship with the serum HDL and any dietary deficiency of choline can lead to blood scarcity of phospholipids and hence HDL. Higher levels of acyl-alkyl-phosphatidyl choline (except diacyl-alkyl-phosphatidyl cholines) also correlated with reduced TG blood level and with improved insulin sensitivity. However, the shorter chain and saturated phosphatidylcholines are positively associated with the risk of T2DM and longer chain unsaturated phosphatidylcholines are protective against it (Table 1) [63]. Wang-Settler et al. [4] also identified low levels of LysoPC 18:2 and glycine as an early indicators of the onset of T2DM in a prospective crested case-controlled study. Suhre et al. [64,65] also identified numerous glycerophospholipids associated with the T2DM risk in the KORA F3 case-controlled cohort study. The PCs (34:4, 36:3, 38:5, and 40:1) and LysoPC (20:4) are negatively associated with diabetes. On the other hand, Pes (34:2, 36:2 and 38:4) of the same carbon chain lengths increased in diabetic subjects. The METSIM (Metabolic Syndrome in Men), a prospective population cohort study, adopted a global lipidomic profiling approach and found elevated levels of one PL cluster (LC8), 4 TG clusters (LC9 to LC12) and a decreased concentration of ether alkylacyl phospholipids (PL) cluster at baseline in undiagnosed prediabetic-progressors [58]. The dyslipidemia biomarkers (LDL-C, HDL-C, ALT and total TG) have also shown a positive correlation with the TG clusters (LC9 to LC12), whereas a negative correlation with PL cluster (LC5) and arachidonic acid containing PCs. The lipid profile of normal glucose tolerance (NGT)-non-progressors was similar to the prediabetic non-progressors both at baseline and at the end of a five-year follow-up. LysoPC, SM, highly-unsaturated LCTGs and ceramide-containing lipid clusters increased in prediabetic non-progressors compared to healthy-non-progressors [58]. Rhee and his team correlated dyslipidemia and risk assessment of diabetes incidence. A strong association of TG with a single double bond and low carbon number with the risk of T2DM has been witnessed by Rhee and his colleagues [66]. The short-chained monounsaturated TG were linked with the high prevalence rate of diabetes, whereas large carbon number monounsaturated TG were related to a reduced risk of diabetes. The multivariate adjustments of a regression model with LysoPE, PC, SM and diacylglycerols (DAG) retained the same results. A total of 9 analytes were screened after the regression analysis adjustments with respect to age, sex, BMI, fasting insulin, cholesterol and parental history. With each increment in the SD of the odds ratios of these nine metabolites, the prospects of diabetes incidence increased by 1.35–1.94-fold. The acute studies with exercise and even with administration of glipizide, have also shown that short chain unsaturated TG decreased with OGTT and long chain unsaturated TG increased (Table 1) [59,67]. The plasma levels of these TG were further corroborated with the insulin resistance. The TG levels were found to differ abruptly and differently over the course of studies consisting of 12 years. The integration of negative and positive risk factors of TG with relatively higher carbon numbers and unsaturation index improved the prediction ability of the used model [68]. However, it is still the subject of investigation whether these lipids served only as diabetes predictors or also contributed towards the pathogenesis of DM. In addition to TG, the logistic regression-based predicting models were also proposed for identifying and predicting PL-based-biomarkers in the discovery and validation of cohort sets. These models proposed LysoPC (18:2), LysoPC (32:1), LysoPC (34:2e), TG (17:1),TG (50:5) TG (50:1), TG (18:1), TG (54:5), TG (18:2), TG (56:4) and ether lipid PC (42:6e), as validated biomarkers for early diagnosis/prediction of T2DM [58,59]. Similar kinds of outcomes have been reported by the RISC and Botnia cohort studies which described a reciprocal relationship of LysoPC (18:2) with the risk of T2DM. The fasting plasma level of LysoPC (18:2) measured at baseline independently predicted the risk of T2DM with the same power as of 2 h plasma glucose level [69]. The AusDiab cross-sectional cohort study of undiagnosed T2DM patients also showed an increased AUC for 17 lipid risk factors belong to five classes of lipids i.e., diacylglycerols (DG) (DG 16:0/22:5, 16:0/22:6, 16:1/18:0, 16:1/18:1, 16:0/16:0, 18:0/18:1, 16:0/18:0, 16:0/20:4, 14:0/18:1, 16:0/20:3, and 18:0/18:2), TG (14:1/16:1/18:0, 16:1/16:1/16:1), cholesterol esters (CE) (CE 24:1 and CE 22:0), PE 40:6 and dihydroceramide (DHC) (dhCer 18:0). The inclusion of DAG, in addition to TG and Hb1Ac, in the predicting models significantly improved the independent stratification of patients of impaired glucose tolerance (IGT) from the whole population of NGT. The incorporation of DHC, PEe and CE not only represented elevated levels of TG in potential IGT patients but also reflected separated biological processes in prediabetic patients compared to healthy ones [70]. Stahlman et al. [71] also unveiled the increased composition of DG (16:0/22:5, 16:0/16:0 and 16:0/22:6) and triacylglycerol species in the VLDL-C and LDL-C diabetic dyslipidemic women. Another cross-sectional explorative cohort study on age and health matched lean and obese (prone to T2DM) human subjects, disclosed that abundance of six metabolites varied considerably between the lean and obese persons and considered the predictors of body fat mass. The lipid metabolite (PC 42:0) was found to be abundant in the obese subjects whereas PC (32:1), PC 32:0, and PC (40:5) were higher in the lean subjects [57]. The rest of the lipid-based body fat mass predictor metabolites belong to carnitines and have been described in their respective section. The relationship of obesity with T2DM was further dissected by the metabolomics done by the team of Tulipani et al. [72]. This study unveiled the relationship of glycemic impairment with obesity based on the three lysoPC. These three lysoPC i.e., lysoPC (17:0, 18:1, and 18:2), showed a strong inverse correlation with BMI, body weight, hip circumference and waist. The levels of these lysoPC decreased in those obese subjects who were in the highest quartile i.e., more prone to develop diabetes. The serum phospholipids also showed the same nature of relationships with the dyslipidemic biomarkers however, this relationship was less significant than that of lysoPC [72]. Tulipani and his colleagues also described the elevated levels of nonpolar sphingolipids (dihydro)ceramides (d18:0/18:0 and d18:0/22:0), ceramide (d18:1/18:0) and sphingomyelin (18:0) in those human subjects which later developed T2DM. Suhre et al. [64] conducted a fully comprehensive metabolomics study on a subgroup of T2DM diabetic males (55 years old) of the KORA F3 cohort. This study described phosphatidylcholines PC (34:4) and the lysoPC (20:4) in reciprocal relationship with the risk of T2DM whereas PC with PUFA side chains i.e., PC (40:1, 36:3 and 38:5) were found to be in a positive relationship with T2DM. At the same time, PE with the same side chain length i.e., PE (34:2, 36:2 and 38:4) were found increased in the T2DM patients [64]. The individuals with single nucleotide polymorphism (SNP), another greater risk factor for T2DM, also showed elevated levels of non-esterified fatty acids (NEFAs), acylcarnitines (C2 and C3), several SM, lysoPC and PC in rs7903146 risk allele carriers. The list of metabolites which were observed down-regulated includes SM-OH (24:1), lysoPC (16:0, 16:1 and 17:0) [73]. The difference in PC level between different genotype groups was not significant; however, the unsaturated PC were down-regulated significantly in the SNP-transcription factor 7-like 2 (TCF7L2) group [73]. In an attempt to discriminate the human subjects with NGT, pre-diabetes and T2DM, Zeng et al. [74] found five classifiers metabolites, i.e. 20-hydroxy-leukotriene E4, LysoPC 20:4, 5-methoxytryptamine, Endomorphin-1 and LysoPC 20:3 between NGT and pre-diabetic groups. Similarly five other metabolites i.e., iso-valeraldehyde, linoleic acid, LysoPC (18:1), 2-pyrroloylglycine and dityrosine were found to be strong discriminators between the pre-diabetic and diabetic subjects. The plasma level of PC (18:3/20:3) was found to be increased in pre-diabetes in comparison to NGT subjects whereas PC (18:0/18:2 and 16:0/14:0) reduced in pre-diabetes in comparison to T2DM. Various lysoPC species i.e., lysoPC (20:4, 18:3, 20:5 and 20:3) were also found to be decreased in T2DM patients in comparison to pre-diabetes [74]. Another, recently published work found altered classes of glycerophopsholipids, nucleotide and (deoxy) sugars in the large prospective nested case-controlled study in diabetic and non-diabetic patients. Out of >1300 detected metabolites only 34 were found higher in diabetic patients throughout the study period. Among lipid-based classes, only six metabolites (i.e., PC (22:4/dm18:0, O-20:0/O-20:0 and, O-18:0/22:5) as well as LysoPC (16:0)) showed strong association with T2DM risk. The individuals within the highest tertiles of these metabolites were found to be 4-fold more prone to T2DM [75]. Zhao and his colleagues conducted a cohort study on 3665 American Indians (sixty-five 3-generation and 29 two-generation families), which lasted for 5.5 years [45]. Approximately 9.3% of people became diabetic, whereas 7.5% of the population developed impaired fasting glucose during the study duration. The study found new metabolic lipid-based biomarkers which significantly can predict the risk of T2DM. A total of seven metabolites (PC (22:6/20:4), 3S-7-hydroxy-29, 39, 49, 59, 8-pentamethoxyisoflavan (HPMF), MEIR, LDYR, X-490, 2-hydroxybiphenyl (2HBP) and X-1178) were found in significant association with T2DM risk. The 2HBP and m/z ratio 1178.804 (X-1178, unknown) were found in significant positive association with T2DM risk. Whereas the other 5 detected biomarkers (HPMF, PC 22:6/20:4, two peptides Met-Glu-Ile-Arg and Leu-Asp-Tyr-Arg, and metabolite m/z ratio 490.816 X-490) were found in persons with a decreased risk of T2DM [45]. The study conducted by Conor and his colleagues [76] reported similar results as Zhao et al. [45]. This work disclosed 80–89% higher odds ratios with each rise in the SD of 2HBP and X-1178 (unknown metabolite). On the other hand, 32–42% less risk of T2DM incidence was noticed with each SD increase in HPMF, peptides and PC PC22:6/20:4. García-Fontana et al. [77] also segregated the T2DM with CVD, T2DM without CVD, and control healthy human subjects based on four phospholipids metabolites. These four metabolites were PC (16:1(9Z)/2:0), O-12:0/2:0), LysoPC (O-16:0/0:0), and LPE (18:2(9Z,12Z)). These four metabolites, belonging to three different phospholipids classes, were found decreased in concentration in the T2DM patients. While two metabolites, namely LysoPE (18:2(9Z,12Z)) and LysoPC (O-16:0) discriminated diabetic and diabetic with CVD patients. The levels of these metabolites were further decreased in the diabetic with CVD patients compared to only diabetic patients [77]. The consumption of HFD also induced the specific validated biomarker of pre-diabetes which can be used to predict future risk of T2DM. Wigger et al. [78] fed the six diabetic and obese mice models (DBA/2J, C57BL/6J, Balb/cJ, AJ, 129S2/SvPas, and AKR/J) for a period of three months to check the response of varying genetic background to lipid consumptions. In PL, 3 ceramides, 2 lactosylceramides, and 1 dihydroceramide showed a constructive relation with HOMA-IR and fasting insulin levels whilst six-lipid species showed a negative correlation with the insulin sensitivity suggesting these metabolites as early biomarkers of (pre)-diabetes owing to HFD consumption. This targeted ceramide metabolomic intervention disclosed the elevated levels of Cer (d18:1/18:0, d18:1/20:0 and d18:1/22:0) three years before the diagnosis of T2DM. The plasma concentration of dihydroceramide Cer (d18:0) was found increased 9 years ahead of T2DM occurrence. These findings were further validated by the targeted ceramide profiling in plasma of another CoLaus cohort study which also confirmed the elevated plasma Cer (d18:0) levels from baseline in the diabetic group compared to healthy ones [78]. Moreover, the oversupply of saturated fats in sedentary subjects is known to induce the accumulation of ceramides as a result of up-regulation of sphingolipids biosynthetic pathways (Figure 1) [79]. The accumulation of ceramides promoted insulin resistance by down-regulating the activity of glucose uptake facilitator Akt/PKB [80]. Among many ceramides’ species, two recent and independent studies pinpointed the 16:0 ceramide as main component inducing insulin resistance [81]. The plasma lipidomic of HFD-fed diabetic C57BL/6J mice showed a significantly higher 16:0 ceramide level in the study group than in the control group mice [82]. The 16:0 ceramide expression levels were up-regulated in the liver, white adipose subcutaneous tissue, and brown adipose tissue of obese mice compared to lean mice, both on HFD. At the same time, the knockout of 16:0 ceramide in HFD fed C57BL/6J mice significantly improved insulin sensitivity, energy expenditure and glucose homeostasis [83]. The suggested mechanism of ceramide (over)-biosynthesis involves the dysfunctionality of adipose tissues resulting in excess production of fatty acid precursors of ceramides and DAG which in turn activate the protein kinase C (PKC) notorious for halting insulin signaling in muscles and liver. The inhibition of ceramide biosynthesis also promoted the conversion of white adipose tissue to brown adipose tissue and hence improved lipid and glucose metabolism [84]. Of note, ceramides serve as building blocks of complex sphingolipids like glycosphingolipids and sphingomyelins and this involves a complex set of biochemical reactions catalyzed by serine palmitoyltransferase (SPT). The AA serine can be replaced with l-alanine to carry out this reaction which results in the formation of neurotoxic 1-deoxysphingolipids. The levels of 1-deoxysphingolipids were also found elevated in the plasma of pre-diabetic and diabetic patients. On the basis of these findings, Othman et al. [85] found that the 1-deoxysphingolipids (1-deoxysphingosine (1-deoxySO) and 1-deoxysphinganine (1-deoxySA)) can be used as early biomarkers/risk factors of T2DM. The plasma metabolomics of adult Rhesus monkeys fed on high-fat/fructose-diet for a period of 8–66 months also exhibited the same elevated levels of dihydroceramides and ceramides as obese pre-diabetic and diabetic mice [86]. The noted ceramides species which increased enormously in the pre-diabetic/diabetic include 14:0, 16:0, 22:0 and 24:0 compared to controls. Ceramides are believed to be further metabolized into sphingosine (Sph), sphingomyelin (SM), sphingosine-1-phosphate (S-1-P), sphinganine (Sa) and sphinganine-1-phosphate (Sa-1-P) (Figure 1). The plasma level of Sa and Sph were severely elevated in the diabetic monkeys whereas the levels of these metabolites remained unchanged in the prediabetic monkeys compared to healthy control monkeys. S-1-P was also found to be increased in both diabetic/prediabetic groups whereas Sa-1-P remained unchanged [86]. Gangliosides are a downstream products of ceramides and also belong to sphingolipids. The plasma levels of two gangliosides (16:0 and 18:0) and four glucosylceramides (16:0, 22:0, 24:0 and 24:1) were also found to be elevated in both the prediabetic and diabetic groups compared to controls. The Spearman’s correlation analysis showed a negative relationship between the HOMA-IR and total ceramides, deoxy-sphinganine and 14:0, 16:0, 22:0, 24:0 ceramides [86]. Similar results have been reported previously by the Huas et al. [87] who reported the elevated levels of 18:0, 20:0, 24:1 and total ceramide in the type 2 diabetic human subjects. Elevated levels of myristic, palmitic, stearic, linoleic, oleic and arachidonic acids were also pinpointed by the Xu et al. [88], however, Xu and his colleagues mentioned a decreased level of glycerophspholipids in persons in transition from NGT to IFG. This discrepancy in these results might arise due to the inclusion or exclusion of certain covariates which ultimately affected the final results. The plasma lipidome of (diabetic/non-diabetic) cynomolgus monkey also proposed different plasma polar lipids biomarkers for the prediction of T2DM. This study found elevated levels of phosphatidylglycerol (PG) and PC accompanying lowered plasma concentrations of phosphatidylinositol (PI) (PI 38:4, 36:2, 36:3, 34:2), and PE (38:6, 38:5, 38:4 and 36:3) [89].

Gestational diabetes (GDM) is a condition of high-blood-sugar in healthy pregnant women. GDM affects 3–9% of global pregnancies and women with GDM are considered at an increased risk of developing T2DM. The TG, PL and TC are found to increase in the last trimester of pregnancy. In pregnant GDM subjects, the relative levels of TG were higher than the normo-glycemic pregnant women [90]. Lu and his colleagues [91] identified five positively correlated predictor lipid species i.e., TG (48:1), TG (51:1), and PC (32:1) and two negatively correlated i.e., choline ether phospholipid (PCae) (40:4) and PCae (40:3) with post-load glucose levels. After the adjustment of maternal BMI, age and correction of multiple testing, only the PCae (40:4) were found to be significantly associated with GDM [92]. The correlation of TG (48:1), TG (51:1), and PC (32:1) with T2DM has also been reported previously in the Framingham cohort study which described the elevated levels of these three lipid species in diabetic AusDiab subjects [92]. The presence of these three lipid species which possess a single double bond also implies the existence of monounsaturated fatty acids (MUFA) (i.e., palmitoleate and oleate) in the LCMS spectra. In another cohort study, the hepatic formation of palmitoleate and oleate and circulating plasma palmitoleate and oleate levels have been linked to T2DM risk [93]. Recently, Petry et al. [94] also explored the paternally transmitted genotype and maternal lipid metabolomics revealing considerable associations of TG (44:1) with maternally transmitted fetal imprinted alleles affecting the maternal glucose metabolism during pregnancy starting from the end of the first trimester. This lipid was found associated with insulin resistance in the Framingham Offspring Study too [66,68]. The abundance of TG (44:1) around the 15th week of pregnancy was seen as a risk factor for GDM. Furthermore, a strong correlation was found between the HOMA-IR and TG (44:1) in the DISCOVERY cohort study too. Increased serum levels of total fatty acids, TG, linoleic, arachidonic, esterified cholesterol, glycolytic and Krebs cycle metabolites, 1,5-anhydroglucitol, glucose, palmitoleic, FA derivatives, lysophospholipids, taurine-bile acids and docohexaenoic acids were also witnessed in GDM females compared to NGT females [95,96]. The data-driven approach also identifies distinguishing phospholipids i.e., (LysoPC (16:2), PC (36:3), PG (40:5), PC (48:1), LysoPC (18:0), PC (19:0), PC (32:3), LysoPC (16:0), PC (14:1/dm16:0), PE (15:0/dm18:1) PC (34:6), PC (36:1) and LysoPC (17:0)), long-chain/short-chain fatty acids (LCFA/SCFA) among pregnant women with GDM history, women with NGT (control) and women with NGT but in the upper quartile of glycemic distribution. The metabolites of phospholipids and LCFA/SCFA were higher in the control group compared to the group at the upper quartile. Comparing the GDM group with the upper quartile group, 72 unique metabolic features were identified in which 2-oxoglutaramate metabolite was twice as abundant in GDM than those in the upper quartile group [97]. A nested, pair-matched, case-control study on the GDM women of the Study of Women, Infant Feeding and Type 2 Diabetes after GDM Pregnancy (SWIFT) cohort participants successfully developed a prediction model of GDM-to-T2DM transition, with 83% discrimination power (AUC), comprising of a four-structure metabolic signature (a) hexoses, (b) PC (40:5), (c) BCAA, and (d) SM (14:1) (OH) [98]. The GDM pregnant women of the SWIFT cohort also exhibited lowered levels of PC and sphingolipids than normal healthy controls [99]. Additionally, smaller nested case-controlled study also proposed another validated prediction model consisting of six general dyslipidemic risk factors and three polar lipid metabolites i.e., phosphatidylserine (PS) 38:4, PE (P-36:2) and cholesteryl ester (CE) 20:4 [100]. The univariate receiver operating characteristic (ROC) analysis in a nested, case-controlled, pair-matched study of Asian and Hispanic origin women discovered the elevated levels of fasting triacyglycerlas (TAG) at baseline in those subjects who developed T2DM in a follow up of two years [101]. The multivariate-ROC analysis deliberately exhibited 12 lipid metabolites belong to TAG, ceramide, NEFA, lactosylceramide (LCer), LPC, LPE, PE and SM classes of lipids. The TAG (myristic acid (14:0), palmitic acid (16:0), stearic acid (18:0), oleic acid (18:1), α-linolenic acid, linoleic acid, dihomo-γ-linoleic acid (20:3), eicosapentaenoic acid (20:5) and docosahexaenoic acid (22:5)) levels were increased in the newly diagnosed T2DM females whom progressed from GDM while the rest of lipid metabolites decreased compared to controls. The lactosylceramides (LCer), ceramides and SM which were found decreased in the newly diagnosed T2DM females were LCer (16:0), LCer (24:1), Cer (16:0), Cer (20:0), Cer (22:0), Cer (24:1) and SM (20:1) [101]. These findings of Khan et al. [102] are in contradiction to the previous cohort findings (as described above) in respect to levels of ceramides and other sphingolipids. The reason for this conflict may be due to the inclusion of obesity as a covariate in their statistical analysis while Khan and his colleagues controlled the obesity covariates by pair-matching of BMI in the population. In short, most of PL are considered validated and reliable future risk biomarkers of T2DM. The inclusion and consideration of a positive relationship of short-chained-monounsaturated TG with T2DM also improved the predicting-power of models and came up as unswerving biomarkers. LysoPC and LysoPE were found in a reverse relationship with the occurrence of T2DM except in SNP-diabetes. TG, LCFA, SM, ChoE, and Cer also increased in concentration and showed a direct correlation with the onset of T2DM. In the case of PC, it is difficult to generalize the results for this highly diverse class of PL. Most PC are also increased in the prediabetic state, however, depending on the nature and function of individual PC metabolite, few PC compounds are also found in reciprocal relationship with the risk of PC. The risk factor biomarkers of GDM are different than the prediabetes biomarkers.

Acylcarnitines: acylcarnitines were also found to be potent early reporters of impaired glucose tolerance or otherwise T2DM risk. They are usually generated during the esterification of fatty acids (FA) as per requirement of transporting the FA into mitochondria. The disturbed mitochondrial bioenergetics or mitochondrial stress leads to mitochondrial dysfunctioning which is another phenotype of pre-diabetes leading to a build up of these FA and FA-derived metabolites resulting in incomplete FA oxidation [103,104]. Based on these findings, it was proposed that incomplete oxidation of FA prior to the IR may provide an opportunity to explore novel biomarkers of diabetes. Additionally, carnitines are considered solely responsible for the transportation of LCFA across the inner membranes of mitochondria for β-oxidation and incomplete oxidation of these carnitines produces an intermediated carnitines oxidation called acylcarnitines [103]. Malonyl-CoA, a precursor of malonylcarnitine, plays a significant role in the completion of β-oxidation of fatty acids by inhibiting the expression of carnitine palmitoyltransferase I. The expression level of Malonyl-CoA was found to decrease in the obese and diabetic subjects leading to incomplete β-oxidation of FA and generating acylcarnitines [105]. Furthermore, cellular lipotoxicity also happened due to excessive accumulations of these partially oxidized FA in livers, muscles, adipocytes andpancreatic β-cells paving the way to IR and loss of pancreatic β-cells function. This recognized pre-diabetic state is followed by the less disintegration of LCFA and blunted oxidation of carbohydrates at a cellular level. This event is further accompanied by the imbalance of accumulating LCFA and cellular oxidative capacity leading towards the pile up of lipid-derived moieties (especially carnitines, ceramides, diacylglycerol) which attenuate the insulin signaling by activating the protein kinase C enzymes, and inhibiting Vakt/PKB murine thymoma viral oncogene homolog/protein kinase B respectively. The perturbation of LCFA catabolism in mitochondria leads to the build up of SCFA which aids in the pro-inflammatory cascades and insulin resistance [35,106].

Elevated plasma/serum circulating levels of several SC acylcarnitines (C2, C3, C4, C5, C6, C8), medium chained acylcarnitines (C10, C10:1, C12, C12:1, C14, C14:1) and LC acylcarnitines (C16, C18, C18:1, C20) have already been reported in the pre-diabetic, glucose intolerant and established diabetic cases [55,104]. Many studies focused on identifying these incomplete oxidized LCFA products to set up the signature biomarkers for IR or T2DM [35,106]. The plasma metabolic fingerprinting of weight/age-matched diabetes/non-diabetic African-American women with/without uncoupling protein 3 (UCP3) (missense polymorphism, g/g or g/a), that reduced the oxidation of LCFA, revealed two acylcarnitines namely glutamate and 2-oxoglutarate (α-ketoglutarate) were found to be substantially increased in diabetic women with a g/g allele. The already perturbed BCAA and cysteine catabolism also contributed to the stressful anaplerotic process related to IR as isoleucine, leucine, valine and cysteine are the precursors of succinyl-CoA and succinate. Concurrent with this concept, enrichment of plasma valine occurred with the reduction of propionylcarnitine accompanying worsening of blood glucose control and plasma accumulation of acetylcarnitines [35]. Adams and his team [106] discretely found 42 acylcarnitines and free carnitine in overweight, obese diabetic and non-diabetic subjects with or without UCP3 g/g or g/a polymorphism. Two carnitines were found in different amounts with respect to genotype in g/g and g/a polymorphic subjects. In diabetic polymorphic subjects, C12 carnitines were higher in g/g subjects compared to g/g diabetic patients. Non-diabetic polymorphic patients showed more variation in butyrylcarnitine with 57% reduced content in g/a participants. The concentrations of lactate carnitines were lowered in both polymorphic non-diabetic and diabetic subjects. Irrespective of genotype factors, total carnitines:carnitine ratios rose up to 150–170% in diabetic patients. Acetylcarnitine was the most abundant carnitine moiety which also rose to 157% in diabetic patients (Table 1). Among the medium-chain (MC) carnitines, the level of C6–C10 carnitines rose to 300% in T2DM patients accompanied by a 36% reduction in propionylcarnitine concentration. The blood level of only one carnitine called propionylcarnitine was found in inverse relation with the glucose level in blood [106]. A cross-sectional prospective cohort study with subjects from Nutrition and Health of Aging Population in China (NHAPC) identified that individuals who developed T2DM in the follow-up of 6 years showed higher baseline plasma concentrations of SC, MC and LC acylcarnitines [107]. A strong correlation has also been found to exist between these (especially LC) acylcarnitines and (baseline) fasting blood sugar, metabolic traits, HbA1c and insulin resistance. Upon classification of acylcarnitines, only LC acylcarnitines have shown a strong association with the future risk of T2DM. The per unit increase in SD of the odds ratios of these acylcarnitines showed a 2.48- to 9.41-fold increase in risk ratio (RR) for the individuals in upper quartile. The results presented by Sun et al. [107] only presented the correlation of baseline acylcarnitines and T2DM in Asian populations so recently these findings were retested and confirmed by the another nested, case-controlled cohort study of the PREDIMED (Mediterranean Diet in the Primary Pprevention of Cardiovascular Disease) framework [108]. This study demonstrated that SC acylcarnitines (C2, C3, C4OH, C5, and C6) and MC acylcarnitines (C16, C12) more firmly predict the future risk of T2DM compared to LC acylcarnitines (C18, C18:1, and C20). Additionally, the correction of *p* and the adjustment of baseline plasma glucose resulted in the strongest positive association of C5 acylcarnitines with the future risk of diabetes. The authors also measured the per annum changes in the acylcarnitines level which were in line with the baseline results. The per unit increase in the SD of C3, C4OH and C5 carnitines increased the risk ratio of future T2DM up to 44% [108]. Another case control study was conducted on the American population (sedentary lean, obese with glucose intolerance and obese with T2DM) targeting only 46 compounds of acylcarnitines. The authors noted an obvious increase of saturated and unsaturated LC acylcarnitines, C14–OH– and C16–OH–CN, and plasma free acylcarnitines both in obese + T2DM and obese + glucose intolerant subjects relative to sedentary lean non-diabetic people. In relation to pre-diabetic and lean non-diabetic individuals, the T2DM people with obesity were found to have increased plasma levels of SC and MC acylcarnitines [109]. The lean diabetic patients were found to have significantly elevated plasma levels of C4– and C6–CN acylcarnitines whereas obese diabetic subjects were found to have higher plasma levels of C4-dicarboxylcarnitine (C4 DC–CN). Regarding gender, two notable exceptions were C3 and C5 acylcarnitines which were found to be higher only in obese diabetic men compared to obese diabetic women [108]. Another two human clinical trials also described the acylcarnitine pattern in obese and lean persons with/without diabetes suggesting short chained acylcarnitines as a reliable biomarker of future risk of T2DM in both sexes [32,106]. But C3 acylcarnitines were increased in obese diabetic subjects compared to C3 acylcarnitines levels in obese but yet non-diabetic subjects [32,106]. The untargeted metabolomics study on the plasma samples of 578 Swedish men recruited under the framework of the Uppsala Longitudinal Study of Adult Men (ULSAM) [60] detected four acylcarnitines raised in the pre-diabetic subjects and among these four acylcarnitines, two MC acylcarnitines (C10, and C12 carnitines) have been found to be involved in the early prediction and pathogenesis of insulin resistance [110]. The findings of another published work found that MC acylcarnitines began to increase before the LC acylcarnitines, therefore, MC acylcarnitines’ altered levels are more potent early signs of mitochondrial dysfunctions and disease progression. Recently, Libert et al. [111] also explored the possible plasma acylcarnitines difference in metabolic-well-but-lean, overall-metabolic-well-but-obese, metabolic-unwell-and-prediabetic and diabetic-obese human subjects. The data of this study clearly mentioned the increased plasma level of SC acylcarnitines and ratio of SC acylcarnitines: total acylcarnitines in overall-metabolic-well-but-obese, metabolic-unwell-and-prediabetic and diabetic-obese human subjects compared to metabolic-well-but-lean. Libert and his colleagues [111] found elevated levels of 3-OH-butyrylcarnitine and 3-hydroxybutyrate in metabolic-unwell-and-prediabetic and diabetic-obese human subjects compared to metabolic-well-but-lean control subjects. A few previous studies also mentioned the increased plasma level of malonylcarnitine or sum of 3-OHbutyrylcarnitine and malonylcarnitine in prediabetic and diabetic subjects [55,107]. It is worth adding here that Zhang and his colleagues also added contradictory results which showed that human subjects with NGT, pre-diabetes and newly diagnosed T2DM could not be differentiated merely on the basis of short-chain and medium-chain acylcarnitines [56]. The authors noted higher concentrations of LC carnitine esters (i.e., carnitine C22, palmitoylcarnitine C16, carnitine C20, 3-OH-hexadecanoylcarnitine C16-OH and carnitine C24) in the newly diagnosed pre-diabetes group and T2DM groups. The concentration of free acylcarnitines was significantly higher in the pre-diabetic (25.33 mmol/L) and newly diagnosed diabetic subjects (25.33 mmol/L) compared to subjects with normal glucose tolerance (20.28 mmol/L) (Table 1). The correlation of serum acylcarnitines with prediabetic states such as impaired glucose tolerance (IGT) and isolated impaired fasting glycaemia (IFG) were differentiated depending on serum concentrations of acetylcarnitine. The serum concentrations of acetylcarnitine (C2), tetradecenoylcarnitine (C14:1) and octadecenoylcarnitine (C18:1), were found to have a positive correlation with the IGT whilst C2, hexanoylcarnitine (C6), octenoylcarnitine (C8:1), decenoylcarnitine (C10:1), malonylcarnitine/hydroxybutyrylcarnitine (C3DC + C4OH), hydroxyhexadecanoylcarnitine (C16OH) and tetradecenoylcarnitine (C14:1), were found in significantly higher levels in the T2DM patients. The two groups IGT and IFG were distinguished by the serum levels of tetradecadienylcarnitine (C14:2), tetradecenoylcarnitine (C14:1) and octadecenoylcarnitine (C18:1) [56]. The authors also correlated body fat with the serum (free) carnitines levels and found that a significant positive correlation exists between body fat and free carnitine and the acylcarnitines (C16:1, C8:1, C6, C5, C4, C3, C2) but negatively with C14:2 and C18 acylcarnitines [55]. Previously, a metabolomics approach was also used to reveal the correlation of body mass fat with various metabolites (especially acylcarnitines) abundance between the healthy lean and healthy obese subjects who also underwent the dietary (hypocaloric diet), bariatric surgery and physical exercise interventions. The targeted serum metabolomics showed glutamine and the C18:1, C18:2, C14:1-OH, and C2 carnitines as varyingly abundant in the serum of the two groups. C3 carnitines (with 6 isoforms) were found to be the most powerful body fat mass related markers which were found to be up-regulated in obese subjects and remained uninfluenced by the exercise intervention. However, the low carbohydrate diet intake following bariatric surgery reduced the expression of C3 carnitines which bespeaks higher expression of C3 in body fat mass. The physical activity resulted in an increase of carnitines in both the lean and obese groups, however, this increase was found to be diminished after 24 h [57].

Urinary metabolomics using MS also revealed that diabetic and obese subjects excrete more urinary LC acylcarnitines in comparison to healthy controls [104]. Van der Kloet et al. [99] also employed a urinary metabolomics approach to differentiate progressive and non-progressive forms of albuminuria. The diabetic patients with progressive forms of albuminuria were found to have higher urinary metabolites from acidic/carboxylic acidic (i.e., benzoic acid, 5-hydroxymethyl-2-furancarboxylic acid, galactonic acid, and hippuric acid), acyl-glycines (i.e., 2-phenylacetoxy-propionyl, glycine, salicyluric acid, and 3-methylcrotonylglycine), acylcarnitines, and tryptophan metabolism metabolite compared to non-progressive forms of albuminuria. Dellow et al. [112] also added that persons with lose glycemic control excrete more urinary acylcarnitines which might be the result of reduced renal absorption or carnitines acylation. Tamamoğullari et al. [102] reaffirmed that serum levels of total and free carnitines were higher in T2DM patients having no complications rather than diabetic patients with diabetic retinopathy.

The role of acylcarnitines in progression from gestational diabetes mellitus (GDM) to T2DM has also been recently studied [103,113]. These studies noted significantly higher plasma levels of SC acylcarnitines in newly diagnosed GDM women. These works also emphasizes on MC acylcarnitines due to their unlearned role in the pathogenesis of GDM to T2DM. These MC acylcarnitines (i.e., octanoylcarnitine (C8-acylC), hexanoylcarnitine (C6-acylC), laurylcarnitine (C12-acylC), decanoylcarnitine (C10-acylC)) were also observed in the newly diagnosed GDM patients compared to NGT subjects [103,113]. Gall et al. [114] registered the decrease in MC acylcarnitines especially decanoylcarnitine in the case of insulin resistance. The population-based KORA cohort study also reported a decreased level of three metabolites namely glycine, acetylcarnitine, LPC (18:2) in GDM women [4]. The transition of GDM to T2DM was also studied in a SWIFT sub-cohort study which further added the increased level of C6 and C8 acylcarnitines in those GDM patients which transit from GDM to T2DM in a follow-up of two years [115]. Anderson et al. [97] used the data-driven approach (free of any hypothesis) and reported a decrease in the LC acylcarnitines (i.e., dodecanoyl-, octanoyl-, decanoyl-, and tetradecanoyl-) in both pregnant women groups with previous GDM history and those found in the upper quartiles of the glycemic index compared to the control group. In short, acylcarnitines are not only reliable early reporting biomarkers of DM, but can also be used to differentiate different states of metabolic syndrome such as pre-diabetic, IR, obese, SNP-diabetic, GDM and IPD. Some of acylcarnitines such as glutamate, propionylcarnitine malonylcarnitine, sum of 3-OH-butyrylcarnitine and malonylcarnitine, 2-oxoglutarate, C3, C5, C4–OH, C12, C14, C14:1, C16, C18:1, C20 carnitines, butyrylcarnitine, 3-OH-hexadecanoylcarnitine, dodecanoylcarnitine, octanoylcarnitine, decanoylcarnitine and tetradecanoyl carnitine can be readily currently employed to diagnose subjects with IFG, T2DM, IR, GDM, IPD, and IGT.

Free fatty acids (FFA): two common origins of circulating FFA are de novo lipogenesis from excessive carbohydrates and cleavage of TG in chylomicrons. The FFA metabolism dysregulation is a key event in the emergence of insulin resistance and Randle et al. [116] proposed the preferential oxidation of FFA over glucose as a major episode leading to metabolic syndrome. The significant correlation of plasma concentration of FFA with dyslipidemia and T2DM was seen for the first time and confirmed in the studies of Jones et al. [117] and Taylor et al. [118]. These studies reported significant alterations in the plasma levels of LCFA between the diabetic and control groups. Later on, some studies also suggested the involvement of altered plasma FFA levels in influencing insulin sensitivity and impaired glucose metabolism. The saturated FA (SFA) including palmitic and stearic acid were found in positive relation with glucose intolerance, impaired insulin sensitivity, impaired insulin secretion and HbA1c. The unsaturated oleic acid was a biomarker of inadequate diabetes control [119]. The lipidomics studies of erythrocytes membranes showed a greater palmitate content (31.1 ± 2.4% in T2DM, 25.4 ± 3.1% in controls, *p* < 0.005) in isolated erythrocyte membranes with a higher SFA/unsaturated FA ratio affecting the fluidity of cells [120]. Grapov and his colleagues [121] investigated the relationship between NEFA and signaling lipids (oxylipins and endocannabinoids) influencing the insulin signaling, inflammation and adipose function. The authors noted a 114% increase in the circulating FFA at baseline which additionally positively correlated with the glucose intolerance in diabetic patients. The net concentrations of SFA (14:0, 16:0, 18:0, 19:0, 20:0), MUFA (16:1n7, 18:1n7, 18:1n9, 20:1n9), PUFA (18:2n6, 18:3n3, 22:4n6, and 22:5n3), and trans-FA (trans 16:1n7, trans 18:2n6) were found to be elevated in T2DM patients. The linoleic acid and α-linolenic acid-derived epoxides also increased by 47–127% and showed a positive correlation with SFA and MUFA in diabetic patients. The arachidonate-derived 14,15- and 11,12-dihydroxyeicosatrieneoates were also increased by 86% in diabetic patients. The OPLS-DA based predictive model also described a shift in the metabolic profile. Four FFA i.e., 18:1n9, 18:0, docosahexaenoyl-ethanolamide (DoHex-EA) and 22:5n6 were the main discriminating FFA between healthy and diabetic individuals. The 18:0 and 18:1n9, and 20:4n6 and 22:5n6 were the early reporters of diabetes related changes in the activity of stearoyl-CoA desaturase and LCPUFA biosynthesis [121]. The Uppsala cross-sectional cohort study found higher serum concentrations of SFA namely palmitic acid (16:0), myristic acid (14:0), and palmitoleic acid (16:1w-7) and reduced serum concentration of omega3/omega6 fatty acid in individuals who develop diabetes over the course of 10 years. The logistic regression based predicting model used in this study identified oleic acid, palmitoleic acid, dihomo-7-linolenic acid and stearic acid as contributors towards hyperglycemia and dyslipidemia [122]. The relationship of individual FFA with T2DM was further elaborated in literature by Wang et al. [123]; Lapolla et al. [124] and Yang et al. [125]. The work of Wang et al. [123] clearly elaborated that the percentage of SFA and MUFA was higher at the baseline in those people who developed T2DM over the course of 9 years in the Atherosclerosis Risk in Communities (ARIC) Study. With respect to individual FA, the findings of Wang et al. [123] are in complete agreement with the previous findings of Vessby et al. [122]. Among the cholestrolemic esterified FA, (16:0), palmitoleic (16:1n-7), and dihomo-α-linolenic (20:3n-6) were found in positive association with incidence of T2DM, whereas in PL, the C16:0 and C18:0 were in positive relationship with the T2DM occurrence [123]. Yang et al. [125] used the PCA non-linear mapping (PCA–NLM) approach and also found FFA i.e., 18:1n9, 12:0, 18:3n3, 18:1n7, 20:5n3, 20:2, 22:5, and 22:6 as early discriminators between potential T2DM patients and healthy controls. Conflicting results have also been reported in the literature stating only even-chained SFA (14:0 to 18:0) in a constructive association with development of diabetes whereas LC SFAs (20:0 to 24:0) and odd chain SFA showed opposite trends [126]. The most comprehensive work in this respect and to also quantitatively relate the FFA with T2DM was published by Tan et al. [127] who used the competitive adaptive reweighted sampling (CARS) method with PLS-DA to seek the most probable biomarkers of diabetes. This study found three FFA i.e., α-linolenic acid (18:3n-3), oleic acid (18:1n-9), and eicosapentaenoic acid (20:5n-3) as the predictive biomarkers of future T2DM risk. The elongation of FFA was also correlated with the insulin resistance and LCFA-elongase (Elov16)-led conversion of palmitate to stearate was found to play a major role in the emergence of pathogenesis of T2DM [128]. The prospective cohort study on healthy/diabetic Uyghur and Kazak Chinese distinguished T2DM individuals from healthy controls were having high 22:6 and 20:4n6, and lower 22:0, 14:1, 18:3n6, and 20:3n6 fatty acids plasma concentrations from baseline [129]. The FFA are also believed to attach with peroxisome proliferator-activated receptors (PPAR) and modulate the transcription factors contributing towards metabolic syndrome. Attaching with the PPAR prompted the proinflammatory responses and impaired endothelial function in non-pregnant healthy subjects [130]. Pankow et al. [131] stated that the correlation of high fasting FFA with the incident of T2DM is independent of sex, waist/thigh ratio, percent body fat, fasting TG concentration, and insulin-mediated glucose uptake. A more recent cohort study reported 3–4 times higher serum FFA levels amongst the newly diagnosed and long time-monitored diabetic patients whereas a moderate correlation was found in the pre-diabetic patients. The authors further recommend the measurement of serum FFA concentration in combination with existing diabetic diagonostic tools to improve the diagnostic accuracy. This study ranked the earlier diagnostic accuracy from the newly diagnosed diabetic patients to those monitored for a long time [132]. Yi et al. [133] demonstrated both esterified and non-esterified FA to screen the DM patients from healthy ones. The esterified FA were mostly storage lipids (cholesterol, TG, PL esters) which are also considered the precursors of non-esterified fatty acid. Taking into consideration other variables (body weight, age and sex), the authors identified nine potential biomarkers (total NEFAs, 24:0, 20:3, 14:0, 18:1n7, 16:1n9, 16:0, 18:1n9, 18:0) to separate the T2DM and healthy individuals. Among these FFA, the coefficients of 16:0, 18:1n9, and 18:0 were quite high in T2DM patients compared to controls [133]. The MCFA, beta-hydroxyisovalerate and arachidonates was lowered in T2DM while LCFA including essential FA linolenate and linolate was elevated in comparison to controls. This study demonstrated decreased PC (hence HDL and total cholesterol) and increased PE (TG) contents in diabetic patients [65]. In line with these results, Gall et al. [114] also found lower levels of multiples glycerophosphocholine species which are highly associated with insulin resistance.

Furthermore, the existing evidence in literature on this topic is still inconclusive and not many studies unequivocally consider the dietary intake of carbohydrates, fats and alcohol influencing de novo lipogenesis of FA. Addressing these issues, some nutritional interventional cohort studies focused on these shortcomings and started to correlate the dietary fat intake with the elevated level of FFA and incidence of diabetes. In a large US prospective Health Professionals Follow-Up Study (HPFS), plasma metabolomics of the participants with the validated self-reported HFD consumptions found 31–52% less risk of incidence of diabetes with the increased plasma concentrations of marker dairy fat FA namely 15:0, 17:0, and *trans*-16:1n-7 [134]. The correlation of dairy fat consumption derived trans-palmitoleate concentrations with dyslipidemic biomarker LDL-C was also investigated in a multi-ethnic cohort study “Multi-Ethnic Study of Atherosclerosis”. The consumption of dairy fat was found to be positively associated with an increased plasma concentration of trans-palmitoleate and this trans-palmitoleate concentration in turn positively correlated with LDL-C and negatively with fasting insulin levels and TG [135]. The increased FFA concentration interferes with the insulin signaling causing peripheral insulin resistance or reduces the accessibility of insulin to skeletal muscles ultimately reducing the glucose transport towards muscles. This lipotoxcity also harms the β-pancreatic cells resulting in the impairment of insulin secretion.

Previously, many studies have also established the relationship of FFA level with the GDM in pregnant women. The experimentally created acute elevation in the FFA caused an increase in insulin resistance in both non-pregnant/pregnant women with/without GDM/T2DM. Elevated blood levels of FFA also resulted in the up to 47% diminution of insulin-stimulated glucose uptake and glycogen synthesis [136]. More recently, the relationship of maternal individual FFA level with GDM and inflammatory response has also been studied [137]. This study indicated a positive relationship of palmitic, stearic, dihomo-γ-linolenic (DGLA), and arachidonic acids with the C-peptide level, cytokines/adipokines, and GDM. In addition to GDM, the other most undiagnosed form of prediabetes is isolated post-challenge diabetes (IPD). The interpretation of T2DM based only on fasting blood sugar fails due to the fact that IPD is often overlooked and misdiagnosed due to its normal fasting glucose levels during the screening process of T2DM, and many authors devised some metabolomics strategies to cover up this gap [138,139]. These studies focused on the FFA profile of such patients and cited concentrations of most of FFA were substantial enough to discriminate between the healthy controls, T2DM and IPD. The concentrations of FFA were lower in healthy controls than T2DM and IPD. Moreover, the three FFA (i.e., 18:1, 18:2 and 18:3) can be used as validated biomarkers for diagnosing the T2DM/IPD subjects from the healthy ones. However, to use 18:2 for screening purposes of T2DM, it is necessary to determine the concentrations of 16:0 and 18:0 too, to differentiate the healthy patients from T2DM. 16:0 can be used to distinguish T2DM and IPD individuals [138,139]. In another study, the authors stated the 15 most significantly varying metabolites among healthy, T2DM, and IPD individuals. The concentration of oleic acid, cholesteryl-β-d-glucoside, linoleic acid, 1,2-distearoyl phosphatidyl serine increased in the T2DM and IPD groups whereas other metabolites, such as lysoPE, DHEA-S, lysoPC, and 5-hydroxykynurenine were considerably lower in both of these groups as compared to healthy individuals. The concentration of three metabolites namely DHEA-S, linoleic acid and oleic acid were found to discriminate IPD and T2DM patients [28].

## 5. Biomarkers of Disturbed Microbiome and Microbiome-Related Metabolites

Organic acids (OA) are produced by intestinal microfloral action on sugars and proteins in addition to glycolysis, lipolysis, gluconeogenesis and AA metabolism. Therefore, it is quite important to also discuss the OA as early biomarkers of diabetes.

OA: human OA metabolites are the compounds with carbon chain length varying from C2 to C6 and majorly produced by the fermentation of carbohydrates and AA. Straight-chain OA are usually produced by the colon-inhibited-microfloral fermentation of starchy and fibrous dietary material whereas branched-chain-OA (BCOA) are produced by bacterial metabolism of AA. The other ways of OA production are the various metabolic pathways i.e., glycolysis, lipolysis, gluconeogenesis and Krebs cycle. Any variations in intestinal microfloras’ compositions usually bring about the changes in the concentrations of plasma/urine/serum/fecal OA which made these short-chain entities tremendous biomarkers of various metabolic syndromes [140]. It is estimated that human beings fulfill their 10% daily energy requirements from these OA [141] and crucial involvement of OA in metabolic procedures (e.g., mitochondrial energy production, nutrient deficiencies, free radical overload, intestinal dysbiosis, and so on) importantly relate them to various biological processes. These gut microflora-produced OA are usually absorbed in the colon to maintain the necessary redox balance and in exchange of bicarbonates. OA are also transported from the lumen to different organs where these compounds act as a substrate and are involved in energy homeostasis signaling including lipids, glucose, and cholesterol metabolism in tissues [142]. There is accumulated evidence in literature proposing the (short-chained)-OA the biomarker for DM and other health conditions [143]. Being the central metabolic regime of energy molecules, Krebs cycle is the first to be perturbed in the case of diabetes-induced enzymatic variation which after all can cause an insufficient recycling of OA. Therefore, many studies also declared OA as a marker of DM, central nervous system diseases, organic acidurias and other metabolic disorders etc. [143,144,145,146].

Three OA namely acetic acid, propionic acid and butyrate are known for their health-promoting activities. The plasma levels of these OA were found to decrease in persons prone to IR, IGT, IFG IPD, obesity and T2DM [147]. Butyrate is a more important reporter of early obesity induced metabolic syndrome in this regard as its decreased concentration was found in obese and pre-diabetic individuals. On the other hand, the increased concentration of butyrate is a symbol of well-being as it suppresses the insulin resistance and weight gain. The increased production of butyrate, by modulation of intestinal micoflora diversity, led to less increased IR and weight gain in HFD-diabetic mice [148]. The inoculation of butyrate producing gut microflora in germ-free-lean mice with metabolic syndrome also showed improved insulin sensitivity [149]. Recently, metagenomic data also supported the fact of a substantial decrease in the butyrate producing gut microbiota in obese mice [150]. The butyrate supplementation in HFD also reduced weight gain and improved insulin sensitivity in obese C57BL/6 mice [142]. Beside butyrate, propionic and acetic acids, patients prone to metabolic syndrome or diabetes were found to have up to a 14-fold higher OA (adipic acid, suberic acid, 3-hydroxyisovaleric acid, 2-hydroxybutyric acid, aceto-acidic acid, 5-hydroxyhexanoic acid, lactic acid, 3-hydroxybutyric acid, fumaric acid, 5-hydroxic hexanoic acid, 2, 3-dideoxypentonic acid) content in their urine due to the phenomenon of generous liberation of FFA from adipose tissue followed by β-oxidation of FA [143,144,146]. Interestingly, an increased concentration of most of these OA suppressed the insulin signaling by activating the free fatty acid receptor 2 (FFAR2) while simultaneously promoting the glucose/lipids metabolism in tissues and preventing the lipid accumulation in adipocyte tissues (i.e., weight loss) [149]. Moreover, immune and adipose tissues have two G-protein-coupled receptors (GPCR) (i.e., GPCR 41 and GPCR 43) for (short chained)-OA which are further linked to G-protein for further downstream targeting. Among the major four classes of G-proteins (i.e., Gi/o, Gs, G12/13, and Gq/11), each class is specified for certain GPCRs which proposed the involvement of SCOA in modulating the response of adipose/immunity tissues [151]. The existence of OA receptors on the surface of immune and adipose tissues suggested the modulatory roles of these metabolites in biological processes. The GPCR41/GPCR43 knockout mice studies showed a huge inflammatory response in various metabolic conditions due to GPCR41/GPCR43-/-immune/adipose cells [152] Whilst the supplementation of butyrate or propionate decreased the mRNA expression levels of inflammatory cytokines GPCR41/GPCR43 knockout mice. The acetate, propionate and butyrate reduced the TNF-α, cytokine and chemokine release from the monocytes and neutrophils while promoting prostaglandin E2 production. These studies concluded that SCOA has a modulatory role in the inflammatory status of obese tissues and thus in IR [153,154].

Han and his team [144] also stated a 0.1–66% increase in plasma contents of 10 OA (acetate, propionate, isobutyrate, 2-methylbutyrate, 3-methylevalerate, isovalerate, valerate, caporate, isocaporate), including three C5 and C6 isomers and two C4 isomers, in T2DM diagnosed patients. The gas chromatography–mass spectrometry (GC-MS)-based metabolomics analysis of serum OA unveiled a higher concentration of glucose intermediates i.e., 3-OH-butyric, lactic acid, adipic, succinic, citric, palmitic, and phenyl acetic acids) in diabetic subjects. The higher excretions of these OA were also cited as an indicator of underlying undiagnosed conditions belonging to metabolic syndrome. The higher excretion of adipic acid, 3-OH-butyric acid and suberic acid were synonymous to the occurrence of ketogenesis and the formation of C6–C8 fatty acids due to beta-oxidation in pre-diabetic patients [155]. The pre-diabetic patients were found to excrete higher levels of 2-OH-butyric, hydroxy-isobutyric acid and 3-OH-butyric acid and low levels of sebacic acid, whereas, obese people only excrete higher amount of suberic acid relative to non-obese and non-diabetic subjects. Higher excretion of hydroxy-isobutyric acid in diabetic patients also indicated a higher fat metabolism resulting in higher serum levels of C4 moieties [155]. Chou et al. [156] carried out the serum metabolomics using the GC-MS platform to discriminate normal, diabetic and IPD patients. This study stated sixteen early biomarkers of diabetes clearly distinguished the individuals at T2DM risk from healthy controls and nine metabolites successfully differentiating the people at risk of diabetes or IPD. Two OA biomarkers namely pyroglutamic acid and α-hydroxybutyrate (α-HB) clearly distinguished the IPD and non-IPD (healthy and T2DM), and healthy vs. IPD individuals, respectively [146,157,158]. β-HB, ethylmalonic acid, and α-HB proved to be signature biomarkers to distinguish between healthy controls and T2DM patients. Moreover, lactate and ethylmalonic acid categorized individuals at IPD and T2DM risk [156,159]. The levels of β-HB and α-HB increased in ascending order from healthy controls to IPD and diabetic patients. The α-HB has also been proposed as a biomarker of the IR and IGT [88]. Most of the detected OA metabolites were identified as TCA cycle intermediates which level was found to fluctuate mostly in diabetes, CKD and CHD. Some studies also cited altered levels of these metabolites in the cardiac and nerve tissues of diabetic persons [160]. Citrate and pyruvate were already declared in perturbed amounts in the urine of diabetic patients. The TCA intermediate succinate was found to be increased in the plasma of patients who developed T2DM [161]. The work of Yuan et al. [146] also defined SCOA (e.g., 4-aminobenzoic acid and oxyl acetic acid) as a predicting biomarker for T2DM risk. Chou et al. [156] also stated pyroglutamic acid as a promising biomarker for distinguishing IPD and healthy non-IPD patients whereas pyroglutamic acid was found decreased in diabetic patients [88]. Sato et al. [162] also observed higher concentrations of isovaleric acids in T2DM patients in a blind, randomized and case-control cohort study. Many studies have documented the higher excretion of other OA (especially uric acid) in diabetic patients compared to healthy controls [140,163,164]. These published results also claimed a higher urine OA being the main causative of uric acid stones and nephrolithiasis in diabetic subjects. The uric acid and sodium retention was also observed in insulin resistance and HOMA-IR model. It also proved a strong relation between high uric acid and the low urine pH and insulin resistance [165]. A low urinary pH can also be a validated marker of future insulin resistance as Abate et al. [166] selected his subjects merely on the basis of the presence of uric acid kidney stones. But later these subjects unveiled less insulin sensitivity and lower urinary pH. Therefore, the patients with low urinary pH are at a stage of increased risk of T2DM development. These studies successfully proposed and defended the so-called obvious correlation between low urinary pH and T2DM/obesity, however, these studies also came up with some major limitations such as ignoring the major dietary, environmental and lifestyle factors in these cross-sectional cohort studies. These factors were partially addressed by Maalouf et al. [167] who provided the fixed metabolic diet to the diabetic patients for the whole study duration. The T2DM patients showed higher net acid excretion (NAE) (NH_4_^+^ + TA) value which symbolized the higher net acid production in diabetic patients. However, this data not only lacks the focus of mechanisms responsible for higher acid production in diabetes but also states urinary anionic measurements being non-significantly different between diabetic and non-diabetic subjects which helped out to formulate the idea of GI alkali loss in diabetic individuals. The accumulation of pancreatic fats which affects the bicarbonates secretion and exocrine activities may result in GI alkali loss in T2DM. Alternatively, some other studies proposed greater intestinal micoflora fermentation due to a delayed transient time in the colon in diabetic patients [168]. A low NH_4_^+^/NAE ratio was also noticed in diabetic patients in some studies [169] with higher TA values in diabetic patients. The impaired NH_4_^+^ secretion might be due to increased FA supply to renal tubules resulting in compromised NH_4_^+^ excretion [167]. The work of Sato et al. [162] also published amazing data about the effects of the administration of different diets and the consequent effects on organic acids in diabetic and non-diabetic patients. The diets rich in saturated fatty acid/total fat negatively interlinked with the organic acid concentration in both groups. Carbohydrates exhibited a positive association with all fecal/plasma organic acids of diabetic patients. A further negative linkage was found between the duration of diabetes and two organic acids named acetic acid and propionic acid. Isovaleric acid was the organic acid least influenced by the dietary habits of diabetic subjects [162]. Conclusively, it is also possible to distinguish the normal, pre-diabetic, IPD, obese and T2DM individuals based on the targeted serum/plasma/urine metabolomics focusing OA as early biomarkers of diabetes and or various stages of diabetes.

Human gut microbiota signature biomarkers: the microbial lives of the whole gastro-intestinal tract are collectively known as gut microflora or gut microbiota. The total number of gastro-intestinal microbiota exceeds 100 trillion which is 3-fold more than the total number of human body cells, which is why the gut environment is considered as a whole functional organ [170]. The five important and predominant phyla of gut microbiome are *Bacteroidetes*, *Actinobacteria*, *Proteobacteria*, *Firmicutes* and *Verrucomicrobia*. The composition of these phyla keeps changing throughout the gastro-intestinal tract depending on various confounding factors such as individual lifestyle, dietary habits, health and physiological factors determining which phenotype would be developed. Regardless of this confounding drawback, gut microbial studies identified some gut microbial signatures as early biomarkers for metabolic syndromes. Moreover, the gut microflora controls many metabolic reactions by the production of many beneficial secondary metabolites (e.g., choline, phenols, bile acids, and SCFAs etc.) involved in the various metabolic signaling pathways. Recent findings have shown that gut microbiota is not only involved in maintaining optimal human health but it is also culpably involved in the pathogenesis of metabolic diseases [150,171,172,173].

Recent studies have focused on unveiling the effect of changed dietary habits on the composition and ecosystem of gut microflora. The consumption of fiber-deficient diets resulted in a lesser extent of fermentation in gut and hence less production of systematic anti-inflammatory short-chain fatty acids (SCFA). These SCFA are important for the synthesis and production of immunoglobulin A and immune-supportive cytokines and failure to produce these health-supporting SCFA results in dysbiosis which is in turn implicated in the increased incidence of diabetes and inflammatory diseases [174]. There have also been reports that the population of health-promoting SCFA-producing gut-bacteria found to have decreased in individuals at risk of developing diabetes [175]. SCFA also promotes the production of glucgone-like-peptide 1 (GLP1) and coheres with G-protein-coupled receptors. The production of GLP1 impedes hepatic gluconeogenesis and glucagon secretion; promotes insulin sensitivity and satiety and hence encourages weight loss. SCOA are another class of secondary metabolites generally produced by the gut microflora. It has been noted that butyrate producing gut-bacteria became less abundant followed by an increase in the population of *Lactobacillus* spp. and *Betaproteobacteria* in obesity and T2DM compared to non-obese control healthy subjects [150,168,171]. The gut microflora population also becomes deficient in the Firmicutes and Clostridia in future T2DM patients as noted by Laresn et al. [168]. The lowering of *Clostridium* in diabetic patients worsens the glucose metabolism since this species is primarily involved in the conversion of primary bile acid into secondary bile acid entities (i.e., cholic and chenodeoxycholic acids) in the large intestine [176,177]. The secondary bile acid entities actually activate the farnesoid X receptor (FXR) and G-protein-coupled receptor 1. The activation of G-protein-coupled receptor 1 ensures the release of GLP-1 which is important for proper pancreatic and hepatic functioning. FXR also controls the glucose metabolism and weight loss maintenance by down-regulating the expression of 6-biphosphatase-1, fructose-1, glucose-6-phosphatase and gluconeogenic phosphoenolpyruvate carboxykinase [177,178]. So a lowered population of gut *Clostridium* is another signal of obesity and perturbed glucose metabolism in glucose intolerant persons. Firmicutes (Gram-positive) and Bacteroidetes (Gram-negative) bacteria comprised almost 90% of gut microflora and some studies proclaimed the ratio of Firmicutes-to-Bacteroidetes as a predictor of dietary habits and hence metabolic disorders linked to these dietary life styles. The obese mice model studies registered a high Bacteroidetes-to-Firmicutes ratio with an increased proportion of *Bacteroidetes* [179]. A positive correlation was also noted between the blood glucose level and the ratios of *Clostridium coccoides*/*Eubacterium rectale*, Bacteroides/Prevotella, and Bacteroidetes/Firmicutes groups. So the lowering of SCFA, SCOA and butyrate producing bacteria, *Firmicutes*, *Clostridia* population are the early biomarkers of T2DM in glucose intolerant patients. Additionally, an increased population of Gram-negative Bacteroidetes and Proteobacteria hinted their role in the pathogenesis onset of T2DM via an endotoxin-led-inflammatory response as lipopolysaccharides and endotoxins were found in higher concentration in their cell membranes [168]. Similarly, the transplantation of fecal microflora from healthy and lean individuals containing the specific gut bacteria into diabetic persons ameliorated the insulin sensitivity in the recipients. This transplantation improved the population of SCFA and SCOA producing strains of bacteria in the insulin-resistant persons [180].

In recent years, human gut microbiome data has been used in conjunction with the metabolome data to comprehensively assess the nutritional and metabolic health status of human beings. Qin et al. [150] did a metagenomic study of gut microflora exploring the metagenome association with T2DM in 345 diabetic + non-diabetic subjects. This metagenome-wide-study showed the enrichment of genes with opportunistic pathogens such as *Clostridium hathewayi*, *Bacteroides caccae*, *Clostridium symbiosum*, *Clostridium ramosum*, *E. coli*, *Eggerthella lenta* in T2DM individuals whilst SCFA, SCOA-producing bacteria, including SS3/4, *Clostridiales* sp., *Faecalibacterium prausnitzii*, *E. rectale*, *Roseburia inulinivorans*, and *Roseburia intestinalis* were found to be enriched in non-diabetic persons. Le Chatelier et al. [181] documented that low bacterial richness/low bacterial gene count favors obesity, insulin resistance and low-grade inflammation and fatty liver. The authors further added that based on the metagenomic analysis of 46 genera of low bacterial gene count and high bacterial gene count, one can distinguish the persons who are more prone to obesity/T2DM compared to those whom are less. Bacterial species such as *Ruminococcus* and *Bacteroides* species which were more dominant in the low bacterial gene count could be early predictors of metabolic disorders such obesity and T2DM whereas dominant species (*Bifidobacterium*, *Faecalibacterium prausnitzii*, *Alistipes*, *Akkermansia* and *Lactobacillus*) of high bacterial gene count are mostly associated with resistance to T2DM. This study supported the idea of a lack of SCFA and SCOA producing bacteria, mucus degrading bacteria and sulfate reducing species (*Desulfovibrio*) in the low bacterial gene count group [181]. Conflicting data were also registered by the European cohort study describing the upregulated abundances of only *A. muciniphila* in individuals with less severe metabolic syndrome, however, this upregulation in the abundance of *A. muciniphila* was concurrent with an increased microbial diversity exhibiting the population-dependent relationship of *A. muciniphila* and T2DM [171]. However, all three of the aforementioned cohort studies unanimously declared the *Lactobacillus* species, *Clostridiales* and SCFA/SCOA-producing bacteria as main early discriminants among healthy, glucose intolerant and diabetic human subjects [150,171,181]. The supplementation of *A. muciniphila* in high-fat diet-induced obese mice also increased high-glucose-tolerance and lowered inflammation by lessening the lipopolysaccharides and lipid oxidation [182]. The skewing of gut microbial population, during the early, glucose intolerant and undiagnosed stages of diabetes, affects the cell-to-cell integrity in the gut lining resulting in a leaky gut with increased permeability leading to perturbed immune response and intestinal inflammation. All these variables attributed to influence the T-cell mediated autoimmunity and related autoimmune disorders including diabetes [183,184]. Zhang et al. [185] also performed the metagenomic study using the 16S RNA and short-gun sequencing method to find the gut microflora signature markers among the healthy, pre-diabetic, and newly diagnosed T2DM patients. The healthy controls were rich in *Haemophilus parainfluenzae* T3T1 and *F. prausnitzii* but less abundant in *A. muciniphila* and *Clostridiales* spp. SS3/4. The butyrate producing *Roseburia intestinalis* and *Faecalibacterium prausnitzii* were found to be deficient in T2DM subjects with the abundance of *Streptococcus mutans*, *Lactobacillus gasseri* and certain *Clostridiales*. A fall in the populations of anti-inflammatory strains of *Faecalibacterium prausnitzii* and *Roseburia* (butyrate-producing *Clostridialis*) were seen as an early sign of T2DM compared to healthy controls whereas enrichment of *Akkermansia muciniphila* strains come along with signs of improved metabolic control in the obese mice [186].

The composition of gut microflora cannot always be synonymous to a function and that is why, recently, some studies extended their investigation by correlating the gut microbiota composition with their gut-microbe-derived metabolomics. These studies found increased BCAA levels in the glucose intolerant patients prone to T2DM and this increased production of BCAA was correlated with the increased prevalence of two bacterial strains i.e., *P. copri* and *B. vulgatus* [187]. Moreover, diet is considered a key player in shaping the relationship of gut microbiota composition and metabolism with the risk factors of T2DM, so many studies have devoted their objective on the effect of specific diet consumption and their consequent effects on gut microbial communities and their metabolites. The effect of macronutrient constituents of diet has been particularly discussed in the literature. The prevalent intake of animal protein diets increased the T2DM related gut microbial signatures (i.e., *Bacteroides* and *Clostridia*) with the decrease of SCFA- and SCOA-producing bacteria e.g., *Bifidobacterium adolescentis*. The comparison of plant-based-protein rich diets and animal-origin-protein rich diets was also studied in detail which pointed to the dominance of *Bacteroides*, *Alistipes* and *Bilophila* (bile-tolerant anaerobes) in the latter group of diet recipients [188]. The consumption of a high protein and low carbohydrate diet also diminished the population of SCOA producing bacteria such as *Roseburia* and *Eubacterium rectale* [189]. The consumption of a high-fat diet also caused an increased in the population of *Clostridia* and *Bacteroides* whereas the intake of low-fat diet resulted in the abundance of SCOA-producing bacteria. The consumption of saturated fat-rich-diet encouraged the setup of *Faecalibacterium prausnitzii* and monounsaturated fat-rich-diet intake reduced the total gut bacterial load [190]. Fish oil-fed mice harbored an elevated population of SCOA (especially butyrate and lactic acid) producing bacteria i.e., *Lactobacillus*, *Streptococcus*, *Verrucomicrobia*, *Bifidobacterium* and *Adlercreutzia* whilst lard-feeding showed an increased population of *Bilophila* and *Bacteroides* [191]. The consumption of sugars (fructose, sucrose, glucose) increased the ratio of *Bifidobacteria*:*Bacteroides*. The addition of lactose with other sugars replicated the same results but with lower population of *Clostridia* species [192]. The consumption of artificial sweeteners produced inverted results with an elevated community of *Bacteroides* [193]. The intake of non-digestible carbohydrate-rich diet (fiber/probiotic) stimulated the community growth of beneficial anti-obesity fermentative commensal microbiota i.e., *Bifidobacteria* and *Lactobacilli* [194] whereas, on the other hand, prebiotics reduced the signature gut microbial biomarkers of obesity and T2DM, i.e., *Clostridium* and *Enterococcus* [195]. The intake of probiotics and prebiotics was found to decrease the risk factors and signature biomarkers of T2DM by stimulating a decrease in blood glucose levels. The supplementation of probiotic strains (*Escherichia coli Nissle 1917*, *Lactobacillus plantarum* 2142, *Lactobacillus rhamnosus* GG) or the supernatant of their spent cultures or their metabolites increased the blood concentration of insulin, insulin sensitivity, improved glucose tolerance and relieved the oxidative stress and oxidative stress-related proinflammatory cytokines [196,197,198]. The administration of probiotics/prebiotics in addition with diet in different mouse models (KK-Ay, NOD, and alloxan-induced-diabetic mice) witnessed the fading of signature T2DM biomarkers i.e., lowered HbA1c, FFA, LDL-H, TG, FBG, and HOMA-IR with subsequent increase in the *Bifidobacterium* and butyrate producing gut microbes [199]. The supplementation of diet with polyphenols of varying origins increased the community size of *Prevotella*, *Bifidobacterium*, *Bacteroides*, *Bacteroides uniformis*, *Enterococcus*, *Blautia coccoides-E. rectale* and *Eggerthella lenta* groups [200,201]. In summary, diminishing intestinal bacterial richness is the first biomarker of undiagnosed metabolic syndrome. Considering the dietary, lifestyle and physical activity covariates, the persistent gut prevalence of certain (pathogenic) microflora (i.e., *Bacteriodetes*, *Clostridium coccoides*, *Proteobacteria*, *Clostridiales*, *Lactobacillus* spp., *Betaproteobacteria*, *Streptococcus mutans*, *P. copri*, *B. vulgatus*, *Lactobacillus gasseri*, *Ruminococcus* spp. *E. coli*, *Eggerthella lenta*) are also early risk predictors for metabolic syndrome and pre-diabetic conditions. Likewise, a decrease in the intestinal population of SCFA and beneficial SCOA are also reporters of a future risk of metabolic syndrome and diabetes.

## 6. Conclusions

The metabolic syndrome and diabetes mellitus are becoming more prevalent in both developed and developing countries. The onset of diabetes can be deferred or even prevented if intervention is accurate at early stages. The existing diagnostic clinical tools are not considered sufficient for early prediction of these conditions. However, several perturbed metabolic biomarkers have been proposed for early prediction of metabolic syndrome. These predictive and risk-factoring metabolic biomarkers have been discovered using high-throughput technologies used in many cohort and predictive modeling (metabolomics) studies. Reported metabolic biomarkers mainly belong to AA, BCAA, SCOA, acylcarnitines, phopsholipids and FFA. These metabolites are considered the intermediate metabolites of carbohydrates, lipids and amino acid-altered metabolism which ultimately distorted the gluconeogenesis, glycolysis, lipolysis, the tricarboxylic acid cycle and proteolysis pathways. These disturbed biomarkers showed significant correlations with elevated blood/plasma/serum glucose level, fasting plasma glucose, insulin resistance, glucose intolerance, HOMA-IR, OGTT and obesity. Moreover, most of these early diabetic biomarkers have been validated and are being used in newly established phenome centers for the purpose of population-based screening. Gut microbes have also been found to be influenced by (the early stages of) T1DM, T2DM and obesity. With some exceptions, the abundance of Firmicutes, Actinobacteria, SCOA-producing-bacteria, Bifidobacteriaceae (Actinobacteria) and *Clostridium* phyla was found to be lower in metabolic syndrome, obese and diabetic persons whereas the population of Bacteroidetes, *Lactobacillus* spp., Rikenellaceae, Proteobacteria, *A. muciniphila* and *Desulfovibrio* spp. was found to be richer in metabolic syndrome, obese, and diabetic individuals compared to healthy subjects.

## Figures and Tables

**Table 1:** A comprehensive list of altered metabolites of lipids and amino acid (AA) origins found in cohort studies mentioned in the respective sections.

Sr. no.	(Parent)-Class of Compound	Metabolites	Nature of Variation	Source	Associated Pathway
**1**	Branched-chain-amino acids/Amino acids (BCAA/AA)	*N*-Acetylaspartate	↓	plasma/urine	Alanine and aspartate metabolism
**2**		Phosphocreatine	↑	plasma/urine	Creatine biosynthesis and amino acid
**3**		Creatinine	↑	plasma/urine	Metabolism, glycine, serine and threonine
**4**		Glycine	↓	plasma/urine	Metabolism
**5**		Guanidinoacetate	↑	plasma/urine	AA metabolism
**6**		Butyrylglycine	↓	plasma/urine	FA metabolism
**7**		Caproylglycine	↓	plasma/urine	Fatty acid metabolism
**8**		*N*-Acetylglutamate	↑	plasma/urine	Glutamate pathway (link with urea cycle)
**9**		Choline	↑	plasma/urine	Glycine, serine and threonine metabolism
**10**		Threonine	↑	plasma/urine	AA metabolism
**11**		Valerylglycine	↓	plasma/urine	AA metabolism
**12**		Alanine	↑	plasma/urine	Glycolysis, alanine and aspartate metabolism
**13**		2-Oxoadipate	↑	plasma/urine	Lysine degradation
**14**		Lysine	↑	plasma/urine	biosynthesis
**15**		Glutaric acid	↓	plasma/urine	Lysine degradation, fatty acid metabolism
**16**		Methionine	↑	plasma/urine	Methionine metabolism
**17**		Taurine	↑	plasma/urine	Taurine and hypotaurine metabolism
**18**		Tyrosine	↑	plasma/urine	Tryptophan metabolism
**19**		Indoxyl sulfate	↓	plasma/serum	Tyrosine metabolism
**20**		Citrulline	↑	plasma/serum	Urea cycle
**21**		l-Argininosuccinic acid	↑	plasma/serum	
**22**		*N*-Acetyl citrulline	↑	plasma/serum	
**23**		Ornithine	↑	plasma/serum	
**24**		Isobutyrlglycine	↓	plasma/serum	val, leu and ileu degradation
**25**		Isovalerate	↓	plasma/serum	
**26**		Isovalerylglycine	↓	plasma/serum	
**27**		Methylmalonate	↓	plasma/serum	
**28**		Valine	↑	plasma/serum	
**29**		Glutamylvaline	↑	plasma/serum	dipeptide metabolism
**30**		Gamma-glutamylisoleucine	↑	plasma/serum	*g*-glutamyl metabolism
**31**		3-hydroxybutyrate (BHB)	↑	plasma/serum	ketone bodies degradation
**32**		Phenylacetylglutamine	↑	plasma/serum	Phenylalanine and tyrosine degradation
**33**		Phenylalanine	↑	plasma/serum	Phenylalanine and tyrosine degradation
**34**		Homocitrulline	↑	plasma/serum	Urea cycle
**35**		Phenylacetylglutamine	↑	plasma/serum	Dipeptide
**36**		Glutamylvaline	↑	plasma/serum	Saturated fatty acids
**37**		Gamma-glutamylisoleucine	↑	plasma/serum	*g*-glutamyl metabolism
**38**		*N*-acetylalanine	↑	plasma/serum	BCAA metabolism
**39**		Cysteine	↓	plasma/serum	Amino-sugars metabolism
**40**		Leucine	↑	plasma/serum	AA metabolism
**41**		2-ketoisocaproic acid and 2-hydroxybutanoic	↑	plasma/serum	Leucine and methionine metabolism
**42**		cystine	↑	plasma/serum	AA metabolism
**43**		Histidine	↑	plasma/serum	AA metabolism
**44**		Lysine/serine/aspergine	↓	plasma/serum	AA metabolism
**45**		5-l-Glutamyl-taurine	↑	Urine	AA metabolism
**46**		4-Oxoproline	↑	Urine	AA metabolism
**47**		l-Valine	↑	Urine	AA metabolism
**48**		*N*-formylproline	↑	Urine	AA metabolism
**49**		*N*-(3-hydroxybenzoyl)glycine	↑	Urine	AA metabolism
**50**		3-Hydroxyphenylacetic acid	↑	Urine	AA metabolism
**51**		Glucuronide compound	↑	Urine	AAmetabolism
**52**		d-Glutamicacid	↑	Urine	Amino acids metabolism
**53**		Glutamine	↓	plasma/serum	Amino acids metabolism
**54**		2-aminoadipic acid	↑	plasma/serum	Tryptophan metabolism
**55**	(Acyl)carnitines	Total carnitine	↑	plasma/serum	Mitochondrial fatty acids metabolism
**56**		Free Carnitine	↑	plasma/serum	
**57**		Acetylcarnitine (C2)	↓	plasma	
**58**		Propionylcarnitine (C3), C14:2 and C18 acylcarnitines	↓	plasma	
**59**		Hexanoylcarnitine (C6), Octanoylcarnitine (C8), Decanoylcarnitine (C10), Myristoylcarnitine (C14)	↑	plasma	
**60**		Malonylcarnitine, Oleoylcarnitine (C18:1)			
**61**		Suberoylcarnitine (C8-dicarb)	↑	plasma	
**62**		Summed C10-C14 acylcarnitines	↑	plasma	
**63**		2-methylbutyroylcarnitine	↑	plasma	
**64**		3-dehydroxycarnitine	↑	plasma	
**65**		Butyrylcarnitine (C4)	↓	plasma	
**66**		Isobutyrylcarnitine	↑	plasma	
**67**		Valerylcarnitine	↑	plasma	
**68**		Isovalerylcarnitine	↑	plasma	
**69**		3-Hydroxy-isovalerylcarnitine	↑	plasma	
**70**		3-Methyl-crotonylcarnitine	↑	plasma	
**71**		Hexanoylcarnitine (C6)	↑	plasma	
**72**		Phenylacetylcarnitine	↑	plasma	
**73**		Phenylpropionylcarnitine	↑	plasma	
**74**		4-Phenyl-butyrylcarnitine	↓	plasma	
**75**		4-Methyl-hexanoylcarnitine	↑	plasma	
**76**		Octanoylcarnitine (C8)	↑	plasma	
**77**		cis-3,4-Methylene-heptanoylcarnitine	↑	plasma	
**78**		Decanoylcarnitine (C10)	↑	plasma	
**79**		cis-4-Decenoylcarnitine	↑	plasma	
**80**		cis-3,4-Methylene-nonanoylcarnitine	↑	plasma	
**81**		Lauroylcarnitine (C12)	↑	plasma	
**82**		Myristoylcarnitine (C14)	↑	plasma	
**83**		Linoleoylcarnitine (C18:2)	↑	plasma	
**84**		Adipoylcarnitine (C6-dicarb)	↑	plasma	
**85**		Suberoylcarnitine (C8-dicarb)	↑	plasma	
**86**		C18:2-carnitine	↑	plasma	
**87**		C20-carnitine	↑	plasma	
**88**		C20:4-carnitine	↑	plasma	
**89**		C26-carnitine	↑	plasma	
**90**	Organic acids	Malonate	↑	plasma	Fatty acids metabolism
**91**		Lactate	↑	plasma	Glycolysis
**92**		Acetate	↑	plasma	Glycolysis, ala and asp metabolism
**93**		Valeric acid	↑	plasma	Glycolysis, fatty acid b-oxidation
**94**		Formate	↑	plasma	Glyoxylate and dicarboxylate
**95**		N1-Methylnicotinamide	↑	plasma	Nicotinate, nicotinamide metabolism
**96**		N1-Methylnicotinic acid	↑	plasma	
**97**		Nicotinamide-n-oxide	↑	plasma	
**98**		*N*-Methyl-2-pyridone-5-carboxamide	↑	plasma	
**99**		*N*-Methyl-4-pyridone-3-carboxamide	↑	plasma	
**100**		3-Ureidopropanoate	↑	plasma	Purine metabolism
**101**		Orotate	↑	plasma	Pyrimidine metabolism
**102**		Isocaproyl	↓	plasma	Steroid and hormone production
**103**		(s)-Malate	↓	plasma/serum	TCA cycle metabolism
**104**		2-Oxoglutarate	↑	plasma/serum	
**105**		cis-Aconitate	↓	plasma/serum	
**106**		Citrate	↑	plasma/serum	
**107**		Fumarate	↑	plasma/serum	
**108**		Succinate	↑	plasma/serum	
**109**		m-Hydroxyphenyl propionic acid	↑	plasma/serum	Phenyl alanine metabolism (bacterial)
**110**		m-Hydroxyphenyl propionic acid sulfate	↑	plasma/serum	
**111**		Phenyl sulfate	↓	plasma/serum	
**112**		Hippurate	↑	plasma/serum	
**113**		5-Hydroxykynurenine	↑	plasma	Amino acids metabolism
**114**		3-deoxyarabinohexonic acid	↑	serum	Fatty acid metabolism
**115**		Uronic acid	↑	plasma/serum	Glucose metabolism
**116**		Erythronate	↑	plasma	Amino-sugars metabolism
**117**		Gluconic acid	↑	plasma	Carbohydrate metabolism
**118**		Benzoic acid	↓	plasma/urine	Phenolic metabolite
**119**		Acetic acid	↓	plasma/urine	Carbohydrate metabolism
**120**		Propionic acid	↓	plasma/urine	Carbohydrate metabolism
**121**		Butyric acid	↓	plasma/urine	Carbohydrate metabolism
**122**		Isovaleric acid	↓	plasma/urine	Carbohydrate metabolism
**123**		Valeric acid	↑	plasma/urine	Carbohydrate metabolism
**124**		Succinic acid	↑	plasma/urine	Carbohydrate metabolism
**125**		Formic acid	↑	plasma/urine	Carbohydrate metabolism
**126**		Lactic acid	↑	plasma/urine	Carbohydrate metabolism
**127**		Capric acid	↑	plasma/urine	Carbohydrate metabolism
**128**		Caprylic acid	↑	plasma/urine	Carbohydrate metabolism
**129**		Citrate	↑	plasma/urine	Carbohydrate metabolism
**130**		Ethylmalonic acid	↑	plasma/urine	Carbohydrate metabolism
**131**		Fumarate	↑	plasma/urine	Carbohydrate metabolism
**132**		Glutaric acid	↑	plasma/urine	Carbohydrate metabolism
**133**		Glycolic acid	↑	plasma/urine	Carbohydrate metabolism
**134**		β-Hydroxybutyrate	↑	plasma/urine	Carbohydrate metabolism
**135**		α-Hydroxybutyrate	↑	plasma/urine	Carbohydrate metabolism
**136**		2-Hydroxyisocaproic acid	↑	plasma/urine	Carbohydrate metabolism
**137**		α-Ketoglutarate	↑	plasma/urine	Carbohydrate metabolism
**138**		Lactate	↑	plasma/urine	Carbohydrate metabolism
**139**		Methylmalonic acid	↑	plasma/urine	Carbohydrate metabolism
**140**		Orotic acid	↑	plasma/urine	Carbohydrate metabolism
**141**		Oxalic acid	↑	plasma/urine	Carbohydrate metabolism
**142**		Oxaloacetate	↑	plasma/urine	Carbohydrate metabolism
**143**		Pyroglutamic acid	↓	plasma/urine	Carbohydrate metabolism
**144**		Pyruvate	↑	plasma/urine	Carbohydrate metabolism
**145**		Sebacic acid	↑	plasma/urine	Carbohydrate metabolism
**146**		Suberic acid	↓	plasma/urine	Carbohydrate metabolism
**147**		Succinate	↑	plasma/urine	Carbohydrate metabolism
**148**		Lactate	↓	plasma/urine	Carbohydrate metabolism
**149**		Hippuric acid	↑	plasma/urine	Carbohydrate metabolism
**150**		Indole-3-carboxylic acid	↑	plasma/urine	Carbohydrate metabolism
**151**		Phenyllactic acid	↑	urine	Carbohydrate metabolism
**152**		Glyoxylate	↑	urine	Energy metabolism
**153**		2-Hydroxybutyrate	↑	plasma/urine	Energy metabolism
**154**		3-Hydroxybutyrate	↑	plasma/urine	Energy metabolism
**155**		3-Hydroxy-3-(3-hydroxyphenyl) propanoic acid	↑	plasma/urine	Energy metabolism
**156**		5-Hydroxymethyl-2-furancarboxylic acid	↑	plasma/urine	Energy metabolism
**157**		Benzoic acid	↑	plasma/urine	Energy metabolism
**158**	Free fatty acids	2-Hydroxy-*N*-valerate	↓	plasma/serum	Fatty acids metabolism
**159**		Docosanoic acid	↑	plasma	Free fatty acid synthesis
**160**		2-Hydroxyvaleric acid	↑	plasma	Free fatty acid synthesis
**161**		C12:0	↑	plasma	Lipid metabolism
**162**		C14:0	↑	plasma	
**163**		C15:0	↑	plasma	
**164**		C16:0	↑	plasma	
**165**		C16:1n-9	↑	plasma	
**166**		C16:1n-7	↑	plasma	
**167**		C18:0	↑	plasma	
**168**		C18:1n-9	↑	plasma	
**169**		C18:1n-7	↑	plasma	
**170**		C18:2n-6	↑	plasma	
**171**		C18:3n-3	↑	plasma	
**172**		C18:3n-6	↑	plasma	
**173**		C20:0	↑	plasma	
**174**		C20:1n-9	↑	plasma	
**175**		C20:2n-7	↑	plasma	
**176**		C20:3n-6	↑	plasma	
**177**		C20:4n-6	↑	plasma	
**178**		C20:5n-3	↓	plasma	
**179**		C22:1n-9	↑	plasma	
**180**		C22:4n-6	↑	plasma	
**181**		C22:5n-6	↓	plasma	
**182**		C22:5n-3	↑	plasma	
**183**		C22:6n-3	↑	plasma	
**184**	(Phospho)-lipids	LysoPC 16:0, 18:0	↑	plasma	Phospholipid metabolism
**185**		PE C34:2, PE C36:2, PE C38:4,	↑	plasma	
**186**		DG 16:0/22:5, DG 16:0/22:6, DG 16:1/18:0, DG 16:1/18:1, DG 16:0/16:0, DG 18:0/18:1, DG 16:0/18:0, DG 16:0/20:4, DG 14:0/18:1, DG 16:0/20:3, and DG 18:0/18:2	↑	plasma	Phospholipid metabolism
**187**		LysoPC C17:0, lysoPC C18:1, LysoPC (18:2), LysoPC C20:4, Lyso C 22:6, LysoPC C18:3, LysoPC C20:5, Lyso-PC Lyso-PC C20:C36:3, Lyso-PC C38:5, Lyso-PC 40:1, Lyso-PC C18:2, Lyso-PC C34:3, Lyso-PC C42:5, Lyso-PC C40:6, Lyso-PC C44:5, Lyso-PC C44:4	↓	plasma	Phospholipid metabolism
**188**		phosphatidylinositol (PI) (PI 38:4, 36:2, 36:3, 34:2)	↓	plasma	Phospholipid metabolism
**189**		phosphatidylethanolamine (PE) (PE 38:6, PE 38:5, PE 38:4 and PE 36:3	↓	plasma	Phospholipid metabolism
**190**		Cholesteryl-β-d-glucoside	↑	plasma	Phospholipid metabolism
**191**		Cholesteryl-β-d-glucoside fragment	↑	plasma	Cholesterol metabolism
**192**		1,2 Distearyole phosphatidyle serine	↑	plasma	Cholesterol metabolism
**193**		Lyso PE 18:2, LysoPE (20:0/0:0), LysoPE (20:2/0:0), LysoPE (20:1/0:0)	↑	plasma	Phospholipid metabolism
**194**		TAG 52:1, TAG 50:0, TAG 48:1, TAG 46:1, TAG 44:1 TAG 48:0	↑	plasma	Lipids metabolism
**195**		PC 34:2, PC 40:1, PC 36:3, and PC 38:5	↑	plasma	
**196**		SM 22:0	↑	plasma	Phospholipid metabolism
**197**		TAG 58:10, TAG 56:9, TAG 60:12	↓	plasma	Phospholipid metabolism
**198**		PC 38:6, 18:2, C34:4	↓	plasma	Phospholipid metabolism
**199**		TAG 50:0 + TAG 58:10	↑	plasma	Lipids metabolism
**200**		PC 22:4/dm18:0, PCO-20:0/O-20:0, PCO-18:0/22:5,LysoPCdm16:0	↑	plasma	Phospholipid metabolism
**201**		LysoPCdm16:0	↑	plasma	Phospholipid metabolism
**202**		GlcCer (d18:0/18:0) PC (16:0/O-16:0) PC (O-14:0/18:0)	↓	plasma	Phospholipid metabolism
**203**		diacyl-PC36:1, PC32:1, PC40:5, and PC38:3	↑	plasma	Phospholipid metabolism
**204**		PC (18:2/dm16:0) PC (O-16:0/18:3) PC (O-16:0/18:3)	↑	plasma	Phospholipid metabolism
**205**		PC (P-16:0/18:2)	↑	plasma	Phospholipid metabolism
**206**		glycerophosphorylcholine [M]	↓	plasma	Glycerolipids metabolism
**207**		PC a C20:4 (alt) [B]	↓	plasma	Glycerolipids metabolism
**208**		PC aa (OH, COOH) C28:4	↓	plasma	Glycerolipids metabolism
**209**		PC aa C34:4	↓	plasma	Glycerolipids metabolism
**210**		SM C14:0, C16:1, SM C22:2, SM C18:1, dihydroceramides d18:0/C18:0, d18:0/C22:0, ceramide d18:1/C18:0	↓	plasma	Glycerolipids metabolism
**211**		PE aa C34:2, PE aa C36:2, PE aa C38:4	↑	plasma	Glycerolipids metabolism
**212**		Gangliosides C16:0 and C18:0 and glucosylceramides (C16:0, C22:0, C24:0 and C24:1)	↑	plasma	Lipid/fatty acid metabolism
**213**		PC aa C34:4, PC 34:4	↓	plasma	Glycerolipids metabolism
**214**		arachidonate	↓	plasma	Polyene metabolism
**215**		myristate (14:0), palmitate (16:0), oleic acid, heptadecanoic acid, margarate (17:0), stearate (18:0), 10-heptadecenoate (17:1n7), oleate (18:1n9), linoleate (18:2n6), linoleamide (18:2n6), linolenate (18:3n3 or 6), eicosenoate (20:1n9 or 11), dihomo-alpha-linolenate (20:3n3), adrenate (22:4n6), TG 14:1/16:1/18:0, TG 16:1/16:1/16:1	↑	plasma	FA metabolism
**216**		cholesterol esters (CE) (CE 24:1, and CE 22:0)	↑	plasma	FA metabolism
**217**		2-hydroxypalmitate	↑	plasma	Medium-chain FA metabolism
**218**		2-hydroxystearate	↑	plasma	SFA metabolism
**219**		caproate (6:0), heptanoate (7:0), pelargonate (9:0)	↓	plasma	
**220**		10-undecenoate (11:1n1)	↓	plasma	
**221**		arachidonate (20:4n6)	↓	plasma	
**222**		3-hydroxybutanoic acid (b-hydroxybutryrate)	↑	plasma	Lipid/fatty acid metabolism
**223**		20-Hydroxy-leukotriene E4, 5-methoxytryptamine, Endomorphin-1	↑	plasma	Lipid/fatty acid metabolism
**224**		2-ketoisocaproic acid	↑	serum	Lipid/fatty acid metabolism
**225**		α-hydroxyisobutyric acid	↑	serum	Lipid/fatty acid metabolism
**226**		β-hydroxybutyric acid	↑	serum	Lipid/fatty acid metabolism
**227**		1-monopalmitin	↑	serum	Lipid/fatty acid metabolism
**228**		1-monostearin	↑	serum	Lipid/fatty acid metabolism
**229**		Cholic acid	↑	urine	Lipid/fatty acid metabolism
**230**		Suberic acid	↓	urine	Lipid/fatty acid metabolism
**231**		Glycocholic acid	↑	urine	Bile acid metabolism
**232**		3,4,5-Trihydroxypentanoic acid	↑	plasma/serum	Lipid/fatty acid metabolism
**233**		Galactonic acid	↑	plasma/serum	Lipid/fatty acid metabolism
**234**		2-Hydroxyglutaric acid	↑	plasma/serum	Lipid/fatty acid metabolism

## Abbreviations

1-deoxysphinganine	(1-deoxySA)
2-hydroxybiphenyl	(2HBP)
8-pentamethoxyisoflavan	(HPMF)
adenosine monophosphate-activated protein kinase	(AMPK)
alkylacyl phosphatidylcholines	(PCe)
alkylacyl phosphatidylethanolamines	(PEe)
amino acids	(AA)
area-under-curve	(AUC)
Atherosclerosis Risk in Communities	(ARIC)
branched-chain a-ketoacid dehydrogenase	(BCKD)
branched-chain amino acid aminotransferase	(BCATm)
branched-chain aminotransferase	(BCATm)
branched-chain keto acid dehydrogenase	(BCKDH)
branched-chained AA	(BCAA)
branched-chain-OA	(BCOA)
Ceramides	(Cer)
cholesterol esters	(CE)
cholesterol esters	(ChoE)
cholesteryl ester	(CE)
choline ether phospholipid	(PCae)
combined glucose tolerance	(CGT)
Cooperative Health Research in the Region of Augsburg	(KORA)
coronary artery disease	(CAD)
deoxysphingosine	(1-deoxySO)
diabetes mellitus	(DM)
diabetic nephropathy	(DN)
diabetic retinopathy	(DR)
diacylglycerols	(DAG)
dihydroceramides	(DHC)
Dongfeng-Tongji	(DFTJ)
endogenous glucose production	(EGP)
European Prospective Investigation into Cancer and Nutrition	(EPIC)
False Discovery rate	(FDR)
farnesoid X receptor	(FXR)
fold change	(FC)
Free Fatty Acid Receptor 2	(FFAR2)
Free fatty acids	(FFA)
Gestational diabetes	(GDM)
glucagon-like peptide-1	(GLP-1)
glucose-6-phosphotase	(G6P)
glycated hemoglobin	(HbA1c)
glycerophospholipids	(GPL)
G-protein-coupled receptors	(GPCR)
Health Professionals Follow-Up Study	(HPFS)
High-density-lipoprotein-cholesterol	(HDL-C)
